# Syndecan-4 Is Essential for Development of Concentric Myocardial Hypertrophy via Stretch-Induced Activation of the Calcineurin-NFAT Pathway

**DOI:** 10.1371/journal.pone.0028302

**Published:** 2011-12-02

**Authors:** Alexandra V. Finsen, Ida G. Lunde, Ivar Sjaastad, Even K. Østli, Marianne Lyngra, Hilde O. Jarstadmarken, Almira Hasic, Ståle Nygård, Sarah A. Wilcox-Adelman, Paul F. Goetinck, Torstein Lyberg, Biljana Skrbic, Geir Florholmen, Theis Tønnessen, William E. Louch, Srdjan Djurovic, Cathrine R. Carlson, Geir Christensen

**Affiliations:** 1 Institute for Experimental Medical Research, Oslo University Hospital Ullevål, Oslo, Norway; 2 Center for Heart Failure Research, University of Oslo, Oslo, Norway; 3 Research Institute for Internal Medicine and Department of Cardiology, Oslo University Hospital Rikshospitalet, Oslo, Norway; 4 Department of Cardiology, Oslo University Hospital Ullevål, Oslo, Norway; 5 Department of Medical Genetics, Oslo University Hospital Ullevål, Oslo, Norway; 6 Department of Mathematics, University of Oslo, Oslo, Norway; 7 Bioinformatics Core Facility, Oslo University Hospital, Oslo, Norway; 8 Boston Biomedical Research Institute, Watertown, Massachusetts, United States of America; 9 Cutaneous Biology Research Center, Massachusetts General Hospital, Harvard Medical School, Charlestown, Massachusetts, United States of America; 10 Center for Clinical Research, Oslo University Hospital Ullevål, Oslo, Norway; 11 Department of Cardiothoracic Surgery, Oslo University Hospital Ullevål, Oslo, Norway; Northwestern University, United States of America

## Abstract

Sustained pressure overload leads to compensatory myocardial hypertrophy and subsequent heart failure, a leading cause of morbidity and mortality. Further unraveling of the cellular processes involved is essential for development of new treatment strategies. We have investigated the hypothesis that the transmembrane Z-disc proteoglycan syndecan-4, a co-receptor for integrins, connecting extracellular matrix proteins to the cytoskeleton, is an important signal transducer in cardiomyocytes during development of concentric myocardial hypertrophy following pressure overload. Echocardiographic, histochemical and cardiomyocyte size measurements showed that syndecan-4^−/−^ mice did not develop concentric myocardial hypertrophy as found in wild-type mice, but rather left ventricular dilatation and dysfunction following pressure overload. Protein and gene expression analyses revealed diminished activation of the central, pro-hypertrophic calcineurin-nuclear factor of activated T-cell (NFAT) signaling pathway. Cardiomyocytes from syndecan-4^−/−^-NFAT-luciferase reporter mice subjected to cyclic mechanical stretch, a hypertrophic stimulus, showed minimal activation of NFAT (1.6-fold) compared to 5.8-fold increase in NFAT-luciferase control cardiomyocytes. Accordingly, overexpression of syndecan-4 or introducing a cell-permeable membrane-targeted syndecan-4 polypeptide (gain of function) activated NFATc4 *in vitro*. Pull-down experiments demonstrated a direct intracellular syndecan-4-calcineurin interaction. This interaction and activation of NFAT were increased by dephosphorylation of serine 179 (pS179) in syndecan-4. During pressure overload, phosphorylation of syndecan-4 was decreased, and association between syndecan-4, calcineurin and its co-activator calmodulin increased. Moreover, calcineurin dephosphorylated pS179, indicating that calcineurin regulates its own binding and activation. Finally, patients with hypertrophic myocardium due to aortic stenosis had increased syndecan-4 levels with decreased pS179 which was associated with increased NFAT activation. In conclusion, our data show that syndecan-4 is essential for compensatory hypertrophy in the pressure overloaded heart. Specifically, syndecan-4 regulates stretch-induced activation of the calcineurin-NFAT pathway in cardiomyocytes. Thus, our data suggest that manipulation of syndecan-4 may provide an option for therapeutic modulation of calcineurin-NFAT signaling.

## Introduction

Sustained pressure overload leads to compensatory myocardial hypertrophy and subsequent decompensation and heart failure, a leading cause of morbidity and mortality. Mechanical stimuli, circulating hormones, as well as autocrine and paracrine factors are involved in initiating myocardial hypertrophy. Transduction of mechanical stimuli may involve molecules that bind the extracellular matrix to the cytoskeleton, but exactly which molecules participate is still unknown. Further unraveling of such signaling pathways is essential for defining novel therapeutic targets.

We have previously shown increased myocardial mRNA expression of all four members of the syndecan family and increased protein levels of syndecan-4 in the hypertrophic non-infarcted region of the left ventricle following myocardial infarction [1]. Syndecan-4 is a transmembrane proteoglycan that connects extracellular matrix proteins to the cardiomyocyte cytoskeleton [2], and has been localized to costameres and Z-discs [3], potentially important sites for signal transduction across the membrane [4], [5]. Moreover, syndecan-4 is a co-receptor for several growth factors [6].

Various intracellular signaling pathways have been implicated in transduction of mechanical stimuli during induction of cardiac hypertrophy, including mitogen-activated protein kinase (MAPK) cascades, protein kinase C (PKC)-mediated signaling [7]–[9] and the calcineurin-nuclear factor of activated T-cell (NFAT) pathway [10]. Interestingly, syndecan-4 has been shown to bind and regulate localization and activity of one of these, namely PKC-α [11].

We hypothesized that syndecan-4 acts as a signal transducer in cardiomyocytes responding to an increase in mechanical stress on the myocardium with subsequent activation of pro-hypertrophic signaling. In this study, we show that syndecan-4 is essential for development of concentric myocardial hypertrophy by acting as a scaffolding protein for mechanical stretch-induced activation of the calcineurin-NFAT pathway.

## Results

### Lack of syndecan-4 inhibits development of concentric myocardial hypertrophy during pressure overload

**Figure 1:**
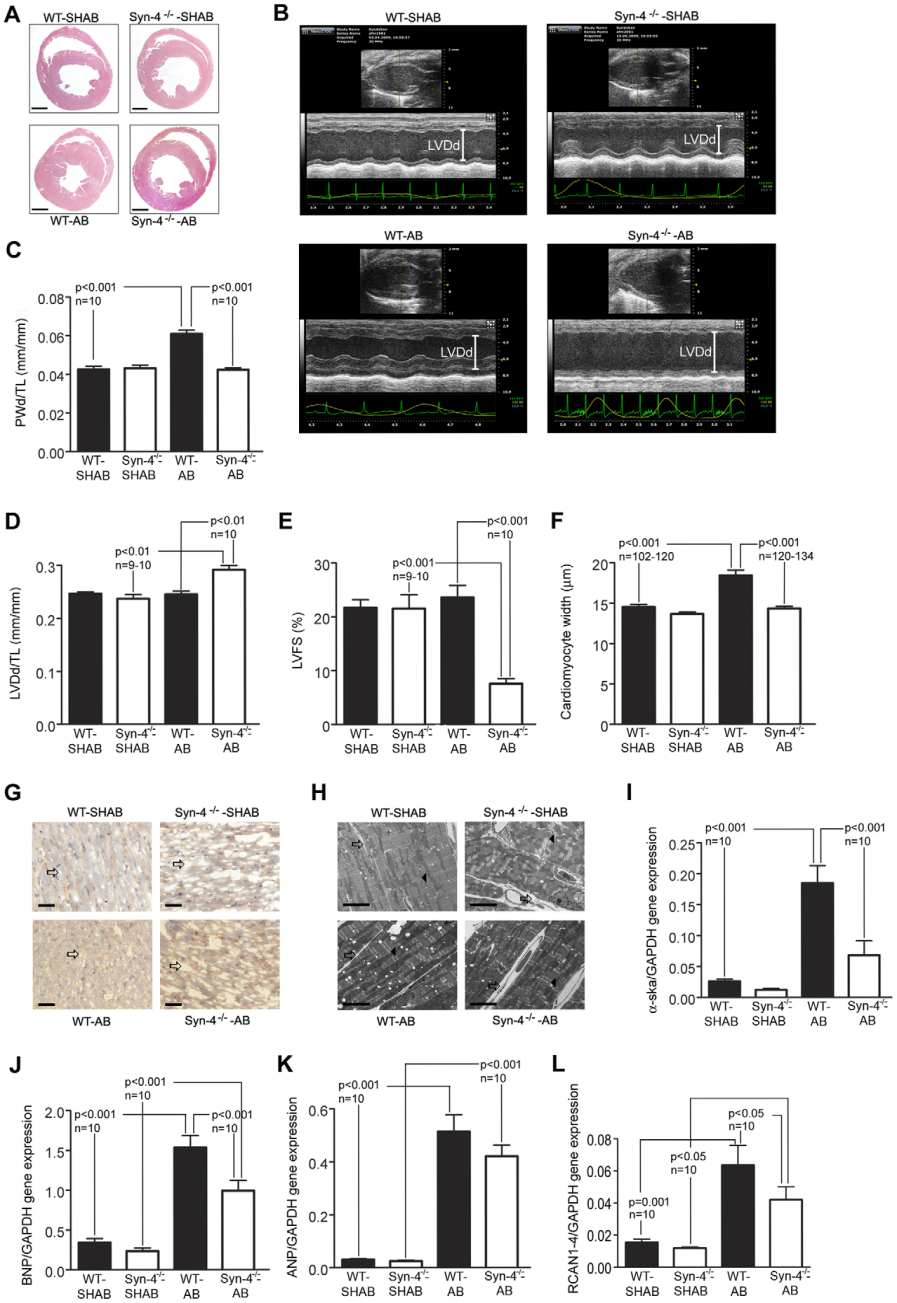
Lack of syndecan-4 inhibits development of concentric hypertrophy three weeks after induction of pressure overload. (A) Representative cross-sections (HE staining) of the hearts of wild-type (WT) and syndecan-4^−/−^ (Syn-4^−/−^) mice after sham operation (SHAB) or aorta banding (AB) (n = 3). Scale bars, 0.1 mm. (B) Representative 2D and M-mode tracings from the left ventricle (LV) (LVDd, LV diameter in diastole) (n = 9–10). Echocardiographic measurements of (C) posterior wall thickness in diastole (PWd) and (D) LVDd normalized to tibia length (TL), and (E) LV fractional shortening (LVFS) (n = 9–10). (F) Cardiomyocyte width (3 animals in each group). (G) Representative sections of LV myocardium stained for collagen I (brown staining indicates collagen I) (n = 2–3). Scale bars, 100 µm. (H) Representative transmission electron microscopy images of LV myocardium (n = 2). Scale bars, 5 µm. Arrows indicate intercellular spaces and arrowheads indicate Z-lines. Real-time PCR analysis of (I) α-skeletal actin (α-ska), (J) B-type natriuretic peptide (BNP), (K) atrial natriuretic peptide (ANP) and (L) regulator of calcineurin 1–4 (RCAN1-4). mRNA levels were normalized to GAPDH in LV (n = 10). Values are mean ± s.e.m.

Syndecan-4^−/−^ mice had normal cardiac dimensions and function compared to wild-type (WT) mice (Table S1). Three weeks after inducing pressure overload by aortic banding (AB), WT developed concentric left ventricular (LV) hypertrophy manifested as a significant increase in posterior wall thickness (Fig. 1A–C). In contrast, syndecan-4^−/−^ mice displayed no increase in wall thickness following AB (Fig. 1A–C), but a significant increase in LV cross-sectional (Fig. 1D) and longitudinal diameter compared to sham-operated syndecan-4^−/−^ mice (syndecan-4^−/−^-SHAB) and WT-AB (Table S1). LV dilatation in syndecan-4^−/−^-AB was paralleled by reduced cardiac function compared to WT-AB, as indicated by reduced LV fractional shortening (Fig. 1E). Accordingly, syndecan-4^−/−^-AB exhibited increased lung weight and left atrial diameter, as well as increased levels of angiotensin II in serum compared to WT-AB (Tables S1 and S2).

In agreement with the observed lack of concentric hypertrophy, cardiomyocyte width was unchanged in syndecan-4^−/−^-AB compared to syndecan-4^−/−^-SHAB (Fig. 1F), whereas in WT-AB cardiomyocyte width was increased by 25% compared to WT-SHAB. The cardiomyocytes were longer in both WT-AB (8%) and syndecan-4^−/−^-AB (10%) compared to respective controls.

LV histological sections demonstrated less densely packed cardiomyocytes in syndecan-4^−/−^ compared to WT after both SHAB and AB (Fig. 1G). This finding was confirmed by transmission electron microscopy, as syndecan-4^−/−^ hearts exhibited wider, irregular gaps between adjacent cardiomyocytes, in addition to disorganized Z-lines, compared to WT following both SHAB and AB (Fig. 1H). Myocardial wet weight was significantly increased in syndecan-4^−/−^ compared to WT only following AB (Table S2). These data demonstrate increased extracellular space in syndecan-4^−/−^-AB compared to WT-AB, in which concentric myocardial hypertrophy with wider cardiomyocytes dominated. Immunohistochemical examination showed equal staining for collagen type I (Fig. 1G), and immunoblotting showed similar changes in collagen I, III and VIII following AB in both genotypes (Table S2).

The lack of cardiomyocyte hypertrophy in syndecan-4^−/−^ mice was associated with lower expression of cardiac markers of hypertrophy (Fig. 1I–J). Expression of α-skeletal actin, previously shown to correlate with myocardial hypertrophy [12] was lower in syndecan-4^−/−^-AB than in WT-AB (Fig. 1I). Expression of B-type natriuretic peptide (BNP), which has been shown to be regulated by the transcription factor NFAT [13], [14] and associated with myocardial hypertrophy, was attenuated in syndecan-4^−/−^-AB compared to WT-AB (Fig. 1J). Expression of atrial natriuretic peptide (ANP) was increased after AB, but there were no differences between the two genotypes (Fig. 1K). Expression of regulator of calcineurin 1–4 (RCAN1-4; also known as MCIP1), a direct target for NFAT [14]–[16], was significantly lower in syndecan-4^−/−^-AB compared to WT-AB (Fig. 1L), indicating reduced NFAT activity in syndecan-4^−/−^ mice after AB. Both the reduction in RCAN1-4 and in BNP are consistent with inhibition of the calcineurin-NFAT pathway, as they have been shown to be direct targets of NFAT in cardiomyocytes [14].

### NFAT signaling is inhibited in syndecan-4^−/−^ mice

**Figure 2:**
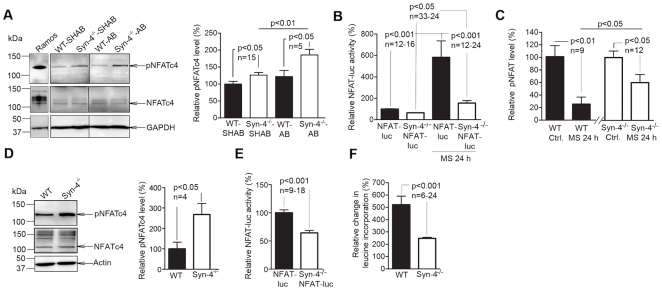
Decreased activation of the calcineurin-nuclear factor of activated T-cell (NFAT) pathway in syndecan-4^−/−^ mice in response to aortic banding. (A) Phosphorylated NFATc4 (pNFATc4) and NFATc4 levels in the left ventricle of wild-type (WT) and syndecan-4^−/−^ (Syn-4^−/−^) mice 24 h after aortic banding (AB) or sham-operation (SHAB) analyzed by immunoblotting. An NFAT-rich cell lysate (Ramos) was used as control for NFAT-positive protein bands (n = 5–15). (B) Luciferase (luc) activity in neonatal cardiomyocytes from NFAT-luciferase and Syn-4^−/−^-NFAT-luciferase mice subjected to 24 h of cyclic mechanical stress (MS) (n = 12–33). (C) pNFATc4 levels in WT and Syn-4^−/−^ neonatal cardiomyocytes subjected to 24 h of cyclic MS (n = 9–12). (D) pNFATc4 and NFATc4 levels in neonatal cardiomyocytes from Syn-4^−/−^ and WT mice subjected to autonomous hypertrophy (n = 4). (E) Luciferase activity in neonatal cardiomyocytes from NFAT-luciferase and Syn-4^−/−^-NFAT-luciferase mice subjected to autonomous hypertrophy (n = 9–18). (F) Radioactive leucine incorporation in Syn-4^−/−^ and WT mice subjected to five days of autonomous hypertrophy (n = 6–24). GAPDH and actin were used for loading control. Values are mean ± s.e.m.

To study the role of syndecan-4 in pro-hypertrophic NFAT activation, NFAT phosphorylation (pNFAT) and NFAT luciferase reporter activity were investigated in pressure overloaded hearts and isolated cardiomyocytes subjected to mechanical stress and autonomous hypertrophy. The calcineurin-dependent NFAT isoform c4 (NFATc4) has previously been found to be sufficient for cardiac hypertrophy [13] and to be activated in the hypertrophic human myocardium [17], and thus activation of this isoform was investigated in our study. Syndecan-4^−/−^ mice demonstrated reduced NFATc4 activation compared to WT following AB, shown as increased pNFATc4 levels (Fig. 2A). Increased levels of pNFAT indicate inhibition of the calcineurin-NFAT pathway, since dephosphorylation of these transcription factors by calcineurin is necessary for translocation to the nucleus. Following sham-operation, there was a minimal reduction in NFAT activation in syndecan-4^−/−^ hearts.

To study whether syndecan-4 acts as a mechanotransducer for activation of calcineurin-NFAT signaling in the pressure overloaded heart, cardiomyocytes were subjected to cyclic mechanical stretch. In NFAT-luciferase reporter cardiomyocytes, 24 hrs of mechanical stretch was sufficient to induce significant calcineurin-dependent NFAT activation (Fig. S1A–B). Importantly, syndecan-4^−/−^-NFAT luciferase cardiomyocytes showed minimal activation of NFAT (1.6-fold) compared to a 5.8-fold increase in NFAT-luciferase control cells following mechanical stretch (Fig. 2B). Accordingly, pNFATc4 levels were significantly higher in syndecan-4^−/−^ cardiomyocytes than in WT cells following mechanical stretch (Fig. 2C). Finally, the role of syndecan-4 in calcineurin-NFAT activation in cardiomyocytes subjected to autonomous hypertrophy, was investigated. Syndecan-4^−/−^ cardiomyocytes subjected to autonomous hypertrophy [18] showed significantly higher levels of pNFATc4 compared to WT cardiomyocytes (Fig. 2D), with a corresponding lower level of NFAT activation as measured by luciferase activity in syndecan-4^−/−^-NFAT luciferase cells (Fig. 2E) and a reduced hypertrophic response as measured by radioactive leucine-incorporation after five days in culture (Fig. 2F). Altogether our data indicate that pro-hypertrophic NFAT signalling was inhibited in cardiomyocytes lacking syndecan-4.

### Overexpression of membrane-localized syndecan-4 *in vitro* activates NFAT

**Figure 3:**
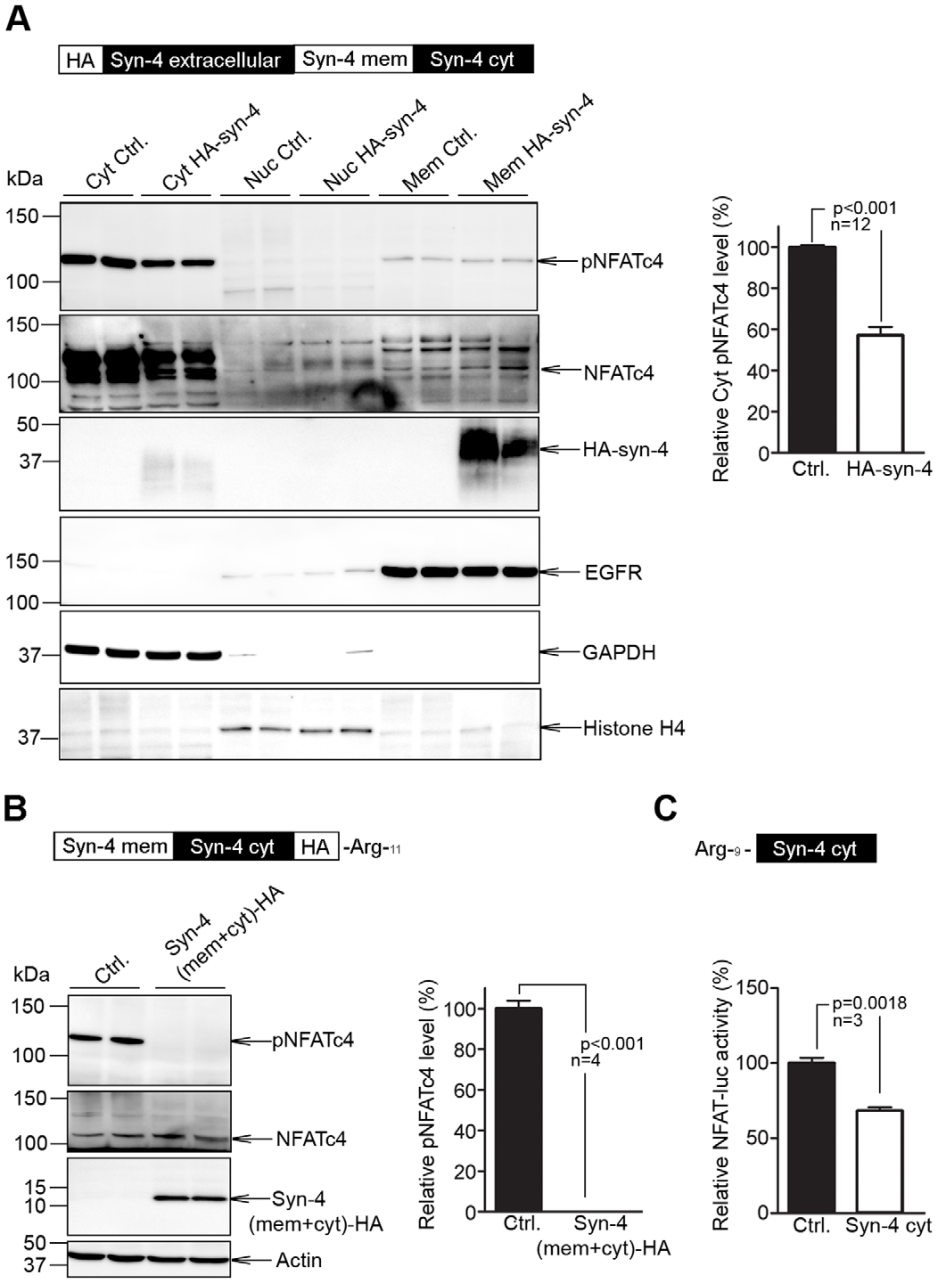
Membrane-localized syndecan-4 activates nuclear factor of activated T-cell (NFAT) *in vitro*. Phosphorylated NFATc4 (pNFATc4), NFATc4 and syndecan-4 levels in (A) HEK-293 cells overexpressing full-length HA-tagged syndecan-4 (HA-syn-4, Cyt = cytoplasmic, Nuc = nuclear, Mem = membrane, EGFR = epidermal growth factor receptor (marker of membrane fraction)) (n = 12). Wild-type (WT) cardiomyocytes treated with a cell-permeable peptide consisting of the membrane and cytoplasmic parts of syndecan-4 (syn-4 (mem+cyt)-HA) (n = 4), analyzed by immunoblotting (B), and NFAT-luciferase reporter cardiomyocytes treated with a cell-permeable peptide consisting of the cytoplasmic part of syndecan-4 only, analyzed by luciferase activity (n = 3) (C). GAPDH, histone H4, EGFR and actin were used as loading controls. Values are mean ± s.e.m.

Increased levels of full-length syndecan-4 in the membrane of HEK-293 cells caused diminished cytoplasmic pNFATc4 levels (Fig. 3A, upper panel) and reduced cytoplasmic/increased nuclear NFAT levels (second panel from top), indicating that increased syndecan-4 levels in the membrane induce NFAT activation by dephosphorylation and translocation to the nucleus. To evaluate syndecan-4 as a regulator of intracellular NFAT activation in cardiomyocytes, we generated a cell-permeable peptide consisting of the membrane and cytoplasmic regions of syndecan-4. Cardiomyocytes treated with this peptide *in vitro* showed complete dephosphorylation of NFATc4, while total amount was unaltered (Fig. 3B), consistent with membrane-localized syndecan-4 being important for NFAT activation through its intracellular domain in cardiomyocytes. To evaluate if membrane localization of syndecan-4 is important for NFAT activation, NFAT-luciferase cardiomyocytes were treated with a cell-permeable peptide consisting only of the cytoplasmic region of syndecan-4. This peptide significantly reduced NFAT-luciferase activity (Fig. 3C), indicating that NFAT activation is inhibited when syndecan-4 is soluble, and not localized in the surface membrane.

We also investigated levels of enzymes that have been reported to regulate NFAT phosphorylation in pressure overloaded hearts, and hence may be involved in the syndecan-4-mediated NFAT activation. There was, however, no difference in the level of calcineurin in WT and syndecan-4^−/−^ hearts after AB (Fig. S2 A). Glycogen synthase kinase (GSK) 3-β phosphorylated by Akt is known to antagonize calcineurin action and development of cardiac hypertrophy by phosphorylating NFAT [19]–[21]. Both GSK3-β and Akt were phosphorylated to the same extent in the two genotypes following AB (Fig. S2 B–D). It has also been shown that each of the three main branches of mitogen-activated kinases (MAPK) directly modulates calcineurin-NFAT signaling and hypertrophic response in the heart [22]. However, activation of extracellular signal regulated protein kinase (ERK) 1/2, c-jun N-terminal kinases (JNK) 2/3 and p38 was comparable in WT and syndecan-4^−/−^ hearts following AB (Fig. S2 E–J). Thus, lack of syndecan-4 did not influence the calcineurin level, GSK-3β or MAPK activation, suggesting that another mechanism regulating NFAT phosphorylation was responsible for the activation of NFAT by syndecan-4. As syndecan-4 is known to directly interact with signaling molecules [23], we hypothesized that syndecan-4 interacts directly with calcineurin.

### Calcineurin associates with syndecan-4, and the association is regulated by dephosphorylation of serine 179 in syndecan-4 during pressure overload

**Figure 4:**
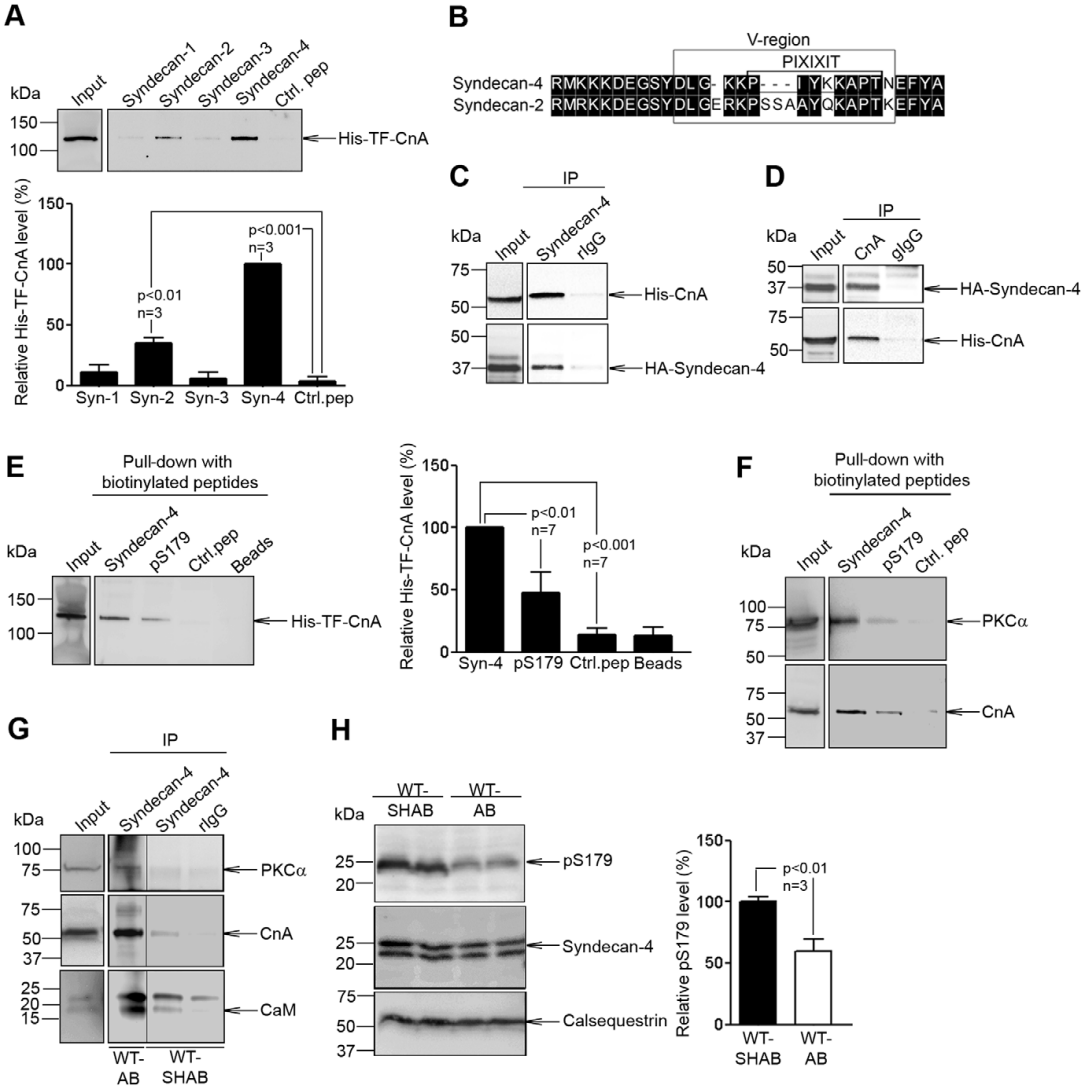
Association of syndecan-4 with calcineurin A (CnA). (A) Pull-down experiments with biotinylated peptides covering cytoplasmic domains of syndecan-1–4 were performed using recombinant His-trigger factor (TF)-CnA, and precipitates immunoblotted for presence of His-TF-CnA (n = 3). (B) Alignment of syndecan-4 and syndecan-2. Black boxes indicate similar amino acids within consensus region. Lysates from HEK-293 cells co-transfected with syndecan-4 and CnA subjected to immunoprecipitation with (C) anti-syndecan-4 (n = 3) or (D) anti-CnA (n = 3) were immunoblotted with anti-CnA or anti-syndecan-4, respectively. Pull-down experiments with biotinylated peptides covering non-phosphorylated or phosphorylated cytoplasmic part of syndecan-4 were performed using recombinant His-TF-CnA (E) or left ventricular (LV) tissue before immunoblotted for presence of endogenous CnA (n = 3) and PKC-α (n = 2) (F). (G) LV tissue extracts from WT-SHAB and WT-AB were immunoprecipitated with anti-syndecan-4 or control IgG and lysates and immunoprecipitates immunoblotted for presence of PKC-α, CnA and calmodulin (CaM, n = 2). (H) Relative pS179-syndecan-4/total syndecan-4 levels in aorta-banded (AB) versus sham-operated (SHAB) WT LV tissue (n = 3). Calsequestrin was used for loading control. Values are mean ± s.e.m.

To examine whether calcineurin associates with syndecan-4, pull-down experiments using biotinylated peptides spanning the cytoplasmic part of syndecan-4 were performed. Recombinant His-trigger factor (TF)-calcineurin subunit A (CnA) was pulled down with syndecan-4 and to a lesser extent with syndecan-2 (Fig. 4A, alignment of syndecan-2 and 4 shown in Fig. 4B). Then, syndecan-4 and CnA were co-transfected into HEK-293 cells and immunoprecipitated with syndecan-4 (Fig. 4C; epitope mapping of syndecan-4 antibodies shown in Fig. S3) and CnA (Fig. 4D) antibodies. Immunoblotting revealed the presence of co-immunoprecipitated CnA (Fig. 4C, upper panel) and syndecan-4 (Fig. 4D, upper panel).

Phosphorylation of serine 179 (pS179) in the intracellular domain of syndecan-4 has been shown to regulate protein interactions, such as PKCα association [24]. Pull-down experiments using biotinylated peptides spanning either the non-phosphorylated or phosphorylated cytoplasmic part of syndecan-4 showed that less recombinant His-TF-CnA bound to pS179-syndecan-4 than to non-phosphorylated syndecan-4 (Fig. 4E). Furthermore, endogenous CnA in LV tissue was pulled down with the non-phosphorylated form, but only minimally with pS179-syndecan-4 (Fig. 4F). As expected, less PKCα bound to pS179-syndecan-4.

In immunoprecipitation experiments using LV tissue, more co-precipitation of CnA with syndecan-4 was observed after AB compared to sham (Fig. 4G). Furthermore, co-immunoprecipitation of the CnA co-activator calmodulin and PKCα were also increased after AB (Fig. 4G). Since pS179 appeared to inhibit syndecan-4-CnA association, pS179-syndecan-4 levels were analyzed following AB using a specific pS179-syndecan-4 antibody. pS179-syndecan-4 levels were found to be reduced by 40% in WT-AB versus WT-SHAB, favoring CnA-syndecan-4 association following AB (Fig. 4H). In the murine heart, syndecan-4 was identified as separate bands at ∼20–24 kDa consistent with reported sizes of syndecan-4 protein [25].

**Figure 5:**
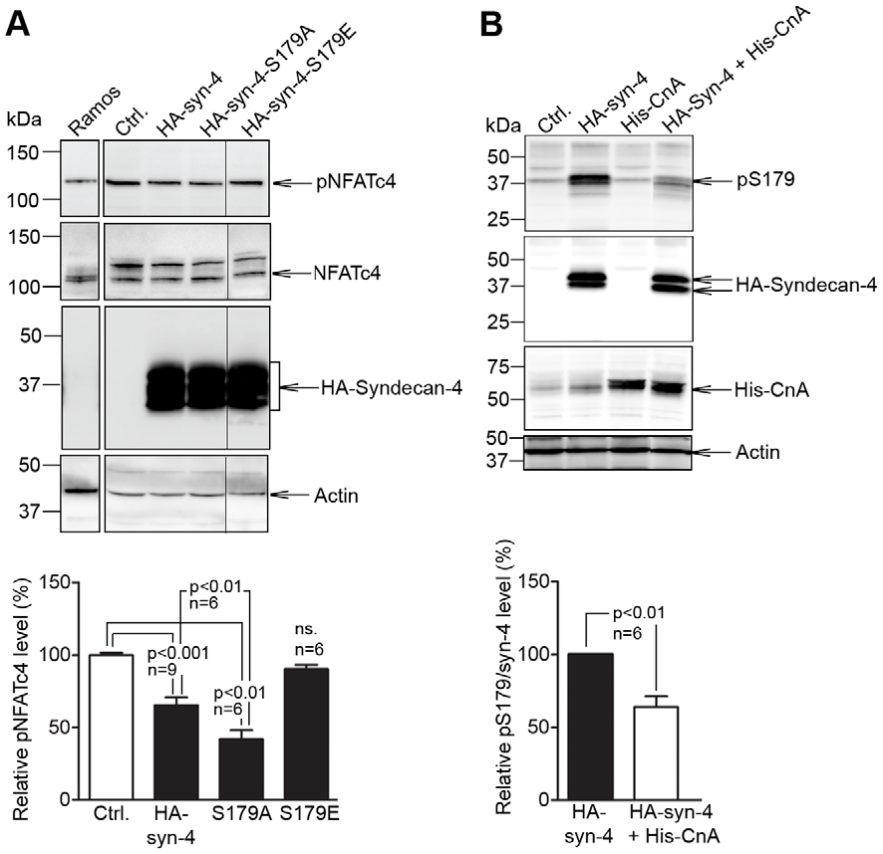
Level of phosphorylated syndecan-4 regulates NFAT activation and is decreased by calcineurin. (A) Phosphorylated NFATc4 (pNFATc4), NFATc4 and syndecan-4 levels in HEK-293 cells transfected with HA-syn-4, HA-syn-4-S179A (mimicking no S179 phosphorylation) or HA-syn-4-S179E (mimicking constitutive S179 phosphorylation) (n = 6–9) and (B) pS179-syndecan-4, syndecan-4 and calcineurin A (CnA) levels in HEK-293 cells co-transfected with CnA and HA-syn-4 or HA-syn-4 alone (n = 6) analyzed by immunoblotting. Actin was used a loading control. Values are mean ± s.e.m.

Consistent with phosphorylation of syndecan-4 being a regulator of CnA association, expression of S179E-syndecan-4 (mimicking constitutive S179 phosphorylation) increased pNFAT levels compared to WT syndecan-4 and was similar to non-transfected cells (Fig. 5A). Conversely, expression of S179A-syndecan-4 (mimicking no S179 phosphorylation) reduced pNFATc4 levels compared to non-transfected cells and WT syndecan-4 expression (Fig. 5A), indicating that membrane-bound syndecan-4 is an activator of CnA.

Finally, co-transfection of syndecan-4 and CnA into HEK-293 cells resulted in reduced pS179 levels compared to syndecan-4 transfection alone, indicating that calcineurin regulates its own binding and activation by dephosphorylation of syndecan-4 (Fig. 5B).

### Intracellular V- and C2-regions in syndecan-4 bind to the autoinhibitory domain of calcineurin

**Figure 6:**
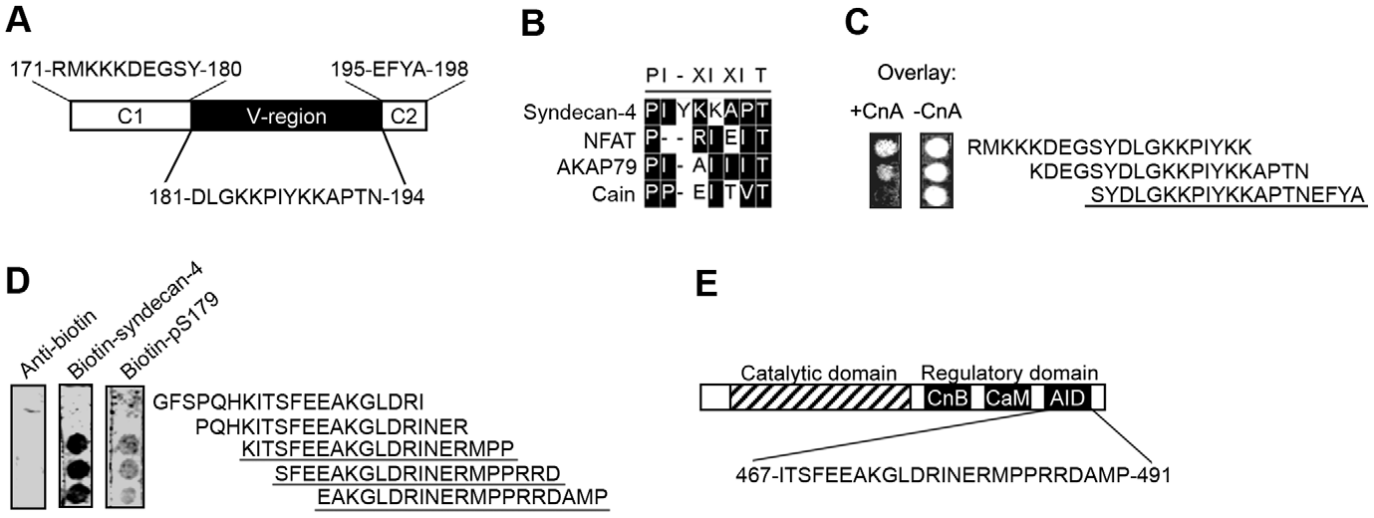
V- and C2-region in syndecan-4 bind to the autoinhibitory domain in calcineurin A (CnA). (A) Schematic illustration of the three cytoplasmic regions in syndecan-4. (B) Alignment of syndecan-4 with other calcineurin binding motifs. Identification of PIxIxIT-similar motif (×represents any amino acid) in V-region. Black boxes indicate similar amino acids within the consensus region. (C) Syndecan-4 residues important for CnA binding were identified by overlaying an array of immobilized syndecan-4 20-mer peptides with 4 amino acids offset with recombinant CnA (left panel). (D) CnA residues important for syndecan-4 binding were identified by overlaying an array of immobilized CnA peptides with 3 amino acids offset with a biotinylated peptide covering the cytoplasmic region of syndecan-4 (middle panel) or the phosphoform (pS179, right panel). Immunoblots without pre-incubation with CnA (C, right panel) or biotinylated peptide (D, left panel) were used as negative controls. Underlined amino acids indicate those relevant for CnA-syndecan-4 binding. Overlay experiments were performed three times with similar results. (E) Schematic illustration of various interaction domains in CnA. Autoinhibitory domain is defined to amino acids 467–491 [53].

The cytoplasmic region of syndecan-4 consists of three regions; two that are conserved between the syndecans, C1 and C2, and the isoform-specific V-region (Fig. 6A). Alignment of syndecan-4 with other CnA-binding proteins; NFAT [26], AKAP79 [27] and calcineurin inhibitor (cain) [28], revealed a putative CnA-binding motif (PIxIxIT) in the V-region (Fig. 6B). The cytoplasmic region of syndecan-4 was synthesized as overlapping 20-mer peptides on a membrane overlayed with active CnA or endogenous calcineurin in tissue lysate followed by immunoblotting. Strongest CnA-binding was observed in the amino acid sequence SYDLGKK**PIYKKAPT**NEFYA, which contains the PIxIxIT–similar motif and the C2-region (Fig. 6C, amino acids 179–198, underlined).

To identify the syndecan-4 binding region, full-length CnA protein was synthesized as 20-mer peptides and overlayed with a biotin-syndecan-4 (171–198) peptide followed by anti-biotin-peroxidase conjugated immunoblotting. Syndecan-4 binding was located to the autoinhibitory domain in CnA (Fig. 6D, middle panel and Fig. 6E). Consistent with our other data, a biotin-phospho-peptide (pS179-syndecan-4) bound only minimally to CnA (Fig. 6D, right panel).

### Increased myocardial syndecan-4 levels and reduced serine 179 phosphorylation in patients with aortic stenosis

**Figure 7:**
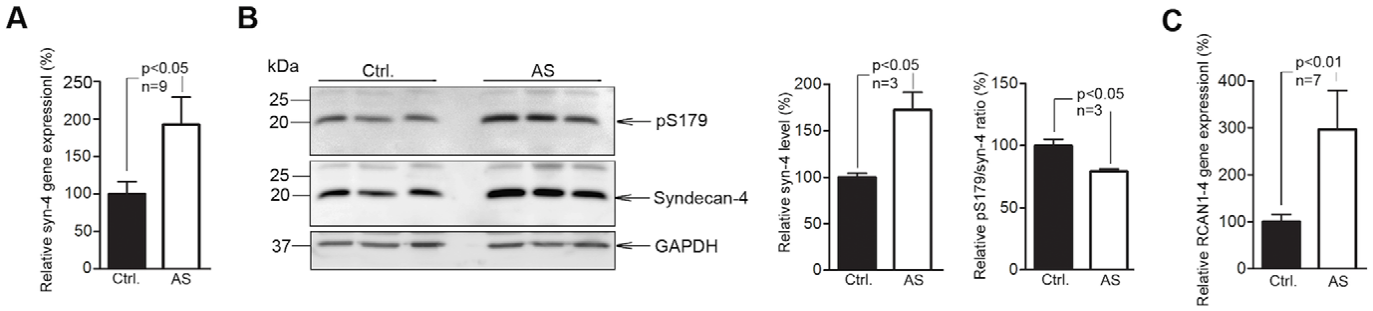
Increased myocardial syndecan-4 levels and reduced serine 179 phosphorylation (pS179) in patients with aortic stenosis. (A) Real-time PCR analysis of syndecan-4 (n = 9), (B) immunoblots of pS179-syndecan-4 (top panel) and syndecan-4 (bottom panel) levels with relative syndecan-4 and pS179-syndecan-4/syndecan-4 ratios (n = 3) and real-time PCR analysis of regulator of calcineurin 1–4 (RCAN1-4) (n = 7) (C) in myocardial biopsies from patients with severe aortic stenosis (AS) versus control patients. GAPDH was used for loading control. Values are mean ± s.e.m.

In order to demonstrate a direct relevance to human heart disease, we examined levels of syndecan-4 and syndecan-4 phosphorylation in biopsies taken peroperatively from patients with hypertrophic myocardium due to aortic stenosis and controls. We found a 92 and 72% increase in syndecan-4 mRNA and protein levels, respectively, in patients with aortic stenosis, with a corresponding 21% reduction in pS179-syndecan-4/total syndecan-4 ratio and a 197% increase in RCAN1-4 mRNA (Fig. 7 A–C), indicating a state that favors calcineurin-NFAT activation in the hypertrophic human heart.

## Discussion

**Figure 8:**
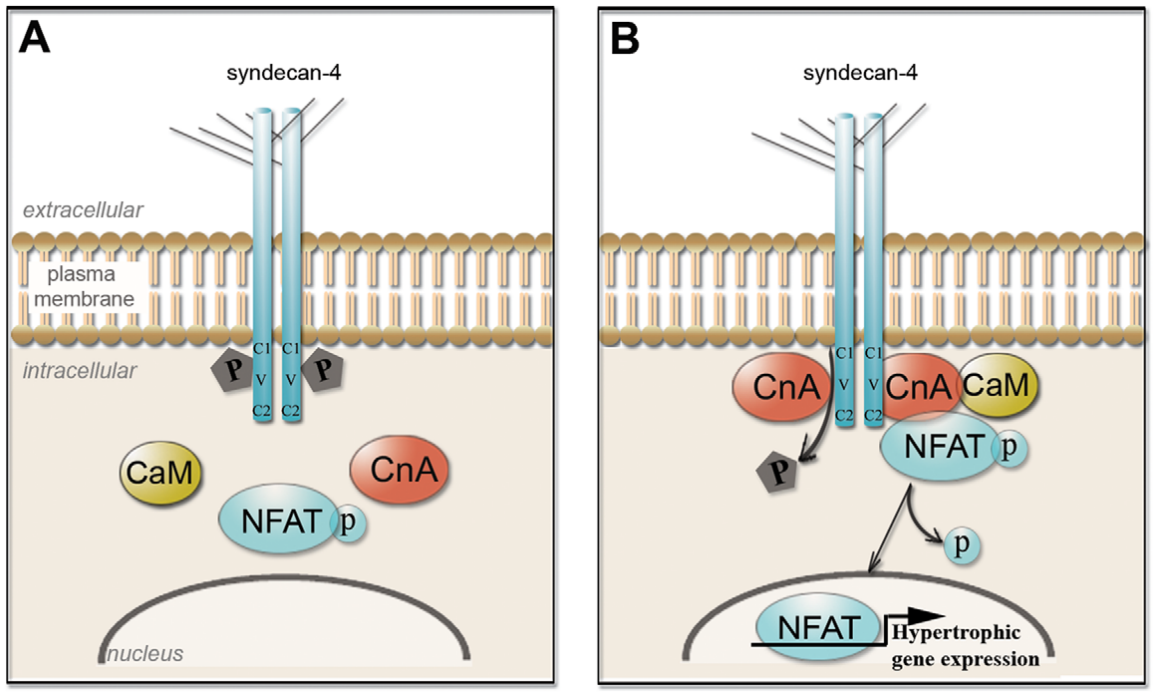
Schematic illustration of proposed syndecan-4-CnA interaction. Phosphorylation of S179 in syndecan-4 renders the CnA-NFAT pathway inactive (left box). Dephosphorylation of syndecan-4 induces binding of CnA and calmodulin (CaM) to syndecan-4 and subsequent activation of the NFAT transcription factor (right box).

Our main findings are that syndecan-4^−/−^ mice did not develop concentric hypertrophy in response to chronic pressure overload, and that lack of syndecan-4 specifically inhibited the calcineurin-NFAT pathway *in vivo* and *in vitro*. In particular, NFAT activation was almost totally inhibited following stretch of syndecan-4^−/−^ cardiomyocytes, whereas it was substantially increased in wild-type cardiomyocytes in response to stretch. Syndecan-4 was shown to bind to the autoinhibitory domain in calcineurin, and this binding was enhanced by dephosphorylation of S179 in syndecan-4 (depicted in Fig. 8). Finally, dephosphorylation of syndecan-4 occurred during pressure overload both in mice and man.

Activation of the calcium- and calmodulin-dependent phosphatase calcineurin is necessary and sufficient to induce pathological cardiac hypertrophy [13], [29]–[32]. Calcineurin dephosphorylates the transcription factors belonging to the NFAT family, leading to their activation [13]. Most studies inhibiting calcineurin activity have produced a significant attenuation of pathological hypertrophy [32], which is in accordance with our *in vivo* findings including lack of concentric hypertrophy during pressure overload in syndecan-4^−/−^ mice.

Signaling molecules involved in the myocardial hypertrophic response to increased mechanical stress, and regulators of the calcineurin-NFAT pathway are only partially known. It has been suggested that mechanical signals in cardiomyocytes converge on membrane proteins such as integrins [33], [34]. In addition, studies indicate a role for mechanotransducer complexes involving the Z-discs and related proteins [35], [36]. As syndecan-4 connects the extracellular matrix proteins to the cytoskeleton, is a co-receptor with α5β1 integrin [37] and is localized to costameres and Z-discs [3], we hypothesized that syndecan-4 senses mechanical stress and activates pro-hypertrophic signaling responses such as the calcineurin-NFAT pathway. A role for syndecan-4 as a mechanotransducer has previously been suggested by us [1] and others [38]. We demonstrate that following stretch, NFAT activation was nearly abolished in cardiomyocytes lacking syndecan-4. We also show that lack of syndecan-4 reduced NFAT activation and expression of the NFAT target gene RCAN1-4 during increased mechanical stress induced by left ventricular pressure overload. These data clearly indicate that syndecan-4 is a regulator of NFAT activity in cardiomyocytes in response to increased mechanical stress.

We then studied the mechanism for activation of calcineurin-NFAT signaling by syndecan-4, and showed that CnA binds directly to the intracellular V- and C2-regions in syndecan-4. The V-region confers binding specificity for each syndecan family member and the syndecan-4 V-region contains a PIxIxIT-similar motif. A loosely conserved PIxIxIT motif has been suggested to represent the CnA binding site in AKAP79 [27], NFATs [26], cain [28], [39] and RCAN1-4 [16], [40]. The syndecan-4 binding region was located to the autoinhibitory domain of CnA. This domain is thought to block the active site within the catalytic region of CnA, rendering the phosphatase inactive [41]–[43]. Increased syndecan-4-calmodulin co-precipitation was observed during pressure overload. Calmodulin has been found to increase calcineurin activity more than 20-fold. Interestingly, the PDZ domain of CASK, which is known to bind calmodulin, is also able to bind to the C2 region of syndecans [44]. Thus, a direct interaction between CnA and syndecan-4 might facilitate activation of calcineurin by bringing calmodulin and CnA in close proximity.

We also demonstrated that CnA-syndecan-4 interaction is regulated by phosphorylation of S179 in syndecan-4. During cardiac pressure overload, we found a decrease in phosphorylated S179-syndecan-4 and an increased association between syndecan-4 and calcineurin, suggesting that dephosphorylation of pS179-syndecan-4 causes increased binding of calcineurin. More directly, we showed that expression of a construct mimicking absent S179 phosphorylation (S179A) increased NFAT activity. These results indicate that alterations in syndecan-4 phosphorylation level may have functional significance for development of cardiac hypertrophy. Moreover, the significantly reduced myocardial phosphorylation of S179-syndecan-4/total syndecan-4 ratios combined with increased NFAT activation found in patients with aortic stenosis indicates a direct relevance for this mechanism in human heart disease. Interestingly, a reduction in phosphorylation of S179 in syndecan-4 was induced by overexpression of CnA, suggesting that calcineurin dephosphorylates syndecan-4 *in vivo* and thereby regulates its own activity.

Concentric hypertrophy of the myocardium during pressure overload is considered to be a compensatory response of the heart to deal with increased afterload during such conditions as aortic stenosis and hypertension. Lack of compensatory hypertrophy may thus cause myocardial dilatation and possibly dysfunction. Accordingly, Meguro et al. [45] showed that inhibition of calcineurin-NFAT activation with cyclosporine attenuated pressure overload hypertrophy and enhanced the susceptibility to heart failure. Similarly, we found reduced cardiac function and attenuated concentric hypertrophy in syndecan-4^−/−^-AB. Although our data clearly indicate syndecan-4-mediated, pro-hypertrophic NFAT activation in cardiomyocytes, syndecan-4 may also mediate other effects in the pressure-overloaded heart. In a recent study, syndecan-4^−/−^ mice were shown to have impaired cardiac function and increased mortality following myocardial infarction [46], while in another study, cardiac function was improved and NFAT activation increased in syndecan-4^−/−^ mice after infarction [47], suggesting additional layers of complexity in syndecan-4-dependent NFAT regulation and remodeling of the heart.

In summary, our data show that syndecan-4 is essential for development of compensatory myocardial hypertrophy in the pressure overloaded myocardium. Specifically, syndecan-4 regulates stretch-induced activation of the pro-hypertrophic calcineurin-NFAT signaling pathway in cardiomyocytes. Syndecan-4 was found to bind to the autoinhibitory domain of calcineurin and this binding was regulated by phosphorylation of syndecan-4. Patients with aortic stenosis exhibited decreased pS179-syndecan-4/syndecan-4 ratios, indicating a direct relevance to human myocardial hypertrophy. In conclusion, our data indicate that syndecan-4 is a scaffolding protein for activation of calcineurin-NFAT signaling and development of concentric myocardial hypertrophy during pressure overload, both in mice and man.

## Methods

The investigation conforms to the Guide for the Care and Use of Laboratory Animals published by the US National Institutes of Health (NIH publication No. 85-23, revised 1996) and was approved by the Norwegian National Animal Research Committee (permit of approval number STFDU1736). The human myocardial biopsy protocol was approved by the Regional Committee for Research.

Ethics in Eastern Norway (permit of approval number 1.2005.316) and conforms to the Declaration of Helsinki.

### Animal model and transthoracic echocardiography

Banding of the ascending aorta was carried out in 7 week old WT (C57BL/6, Møllergaard, Denmark) and syndecan-4^−/−^ mice [48]. Anaesthesia was induced with >5% isoflurane gas and <95% O_2_ in a gas chamber, followed by tracheostomy and ventilation with 2% isoflurane and 98% O_2_, with a tidal volume of 350 µl and a respiratory frequency of 160 min^−1^ on a MiniVent ventilator (Harvard Apparatus, Holliston, MA 01746, USA). A sternal split of the cranial 1/3 of the sternum was performed and an 8-0 silk ligature was tied around the ascending aorta and a 26G blunted needle, which was subsequently removed. Sham-operated animals underwent the same procedure without banding of the aorta. After twenty-one days echocardiography was carried out using a VEVO 2100 (Visualsonics, Toronto, Canada) before the animals were sacrificed. Echocardiographic examinations were performed under standardized conditions with the animals in the supine position, spontaneously breathing 1.5% isoflurane and 98.5% O_2_ on a mask. Echocardiographic data were analyzed off-line using VEVO 2100 1.1.0 software from Visualsonics. Three representative cycles were analyzed and averaged. Two-dimensional (2D) images of the LV were obtained both in long and short axes. Short axis recordings were obtained at the level of the papillary muscle. M-mode tracings were recorded at the level of both the papillary muscles and the aortic valves, with 2D guidance. LV wall thickness and cavity dimensions were measured through the largest diameter of the ventricle both in systole and diastole. LV fractional shortening (LVFS) in percent, was calculated using the following formula.
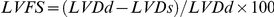
where LVDd is LV diameter in diastole, and LVDs is LV diameter in systole. Doppler recordings were obtained in the left parasternal long axis position. Pulsed wave Doppler was used for measuring flow velocities across the constriction, as well as in the left ventricular outflow tract (LVOT) and in the mitral annulus. Cardiac output (CO) was calculated in LVOT using the following equation
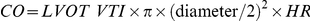
where VTI is the velocity time integral, HR is heart rate and diameter is measured in LVOT.

The LV, right ventricular free wall and lungs were weighed and normalized to tibia length. Only mice with a maximum flow velocity across the stricture >3 m/s and <4 m/s 24 hours after AB and >4 m/s and <6 m/s three weeks after AB were included (mean values, 4.3±0.1 m/s in syndecan-4^−/−^ mice versus 4.3±0.1 m/s in WT).

### LV pressure measurements

Immediately after finishing echocardiography, a 1.4-Fr microtip pressure transducer catheter (SPR-671, Millar Instruments, Houston, TX) was introduced into the left ventricle through the right carotid artery for measurements of LV pressures and calculations of its maximal positive and negative first derivatives. Data recorded from 10–15 consecutive beats were analyzed. LV pressure measurements were performed in un-operated WT and syndecan-4^−/−^ mice, and not following AB as the aortic constriction was too tight to allow for retrograde insertion of the catheter.

### Estimation of LV fluid content

Three weeks after AB or SHAB LV from WT and syndecan-4^−/−^ mice were weighed immediately upon dissection, cut into pieces and dried for 24 h at 90°C, before weighing again.

### Angiotensin II concentration in plasma

Plasma was extracted from full-blood containing 0.44 mM o-phenanthroline, 25 mM EDTA, 1 mM p-hydroxy-mercuribenzoic acid and 0.12 mM pepstatin A and the concentration of angiotensin II was measured using the Max Human Angiotensin 2 ELISA Kit according to the manufacturers protocol (EA3501-1, Assaypro, St. Charles, MO) in WT and syndecan-4^−/−^ mice three weeks after AB or SHAB.

### Human myocardial biopsies

LV apical myocardial biopsies (10 mg) were obtained immediately before crossclamping the aorta in twelve patients with severe aortic stenosis and twelve control patients with coronary artery disease during elective aortic valve replacement and coronary artery bypass operations, respectively. Biopsies were taken from a normal appearing and contracting area using a 22G needle (Easy Core Biopsy System, Boston Scientific International S.A., Cedex, France). Patients 40–85 years old with symptomatic aortic stenosis, a mean aortic gradient >50 mmHg or an aortic valve area <0.7 cm^2^, and without significant coronary artery disease were included in the aortic stenosis group. Patients 40–85 years old with stable angina pectoris, no known previous myocardial infarction, ejection fraction >50%, and without significant valve disease were included in the control group. The patients had no chest pain or electrocardiographic signs suggesting ongoing ischemia preoperatively. Informed written consent was obtained from each patient.

### NFAT-luciferase mice

The NFAT-luciferase mice have nine copies of an NFAT-binding site from the interleukin (IL)-4 promoter (5′-TGGAAAATT-3′) inserted upstream of the luciferase reporter gene, driven by the α-myosin heavy chain promoter [31], and were a kind gift from Jeffery D. Molkentin (Cincinnati Children's Hospital Medical Center, University of Cincinnati, Cincinnati, OH). Neonatal mice from the progeny were used for isolation of cardiomyocytes.

### Syndecan-4^−/−^-NFAT luciferase mice

Syndecan-4^−/−^-NFAT luciferase reporter mice were generated by crossing syndecan-4^−/−^ mice with the NFAT-luciferase mice. Neonatal mice from the progeny were used for isolation of cardiomyocytes.

### Quantification of luciferase activity

For measurements of luciferase activity, cardiomyocytes were harvested according to the Luciferase Assay System protocol (E1500, Promega) and luminescence from sample duplicates was quantified on a Victor 3 1420 Multilabel Counter (PerkinElmer, Waltham, MA).

### Adult cardiomyocyte isolation and cell size measurements

Cardiomyocytes were enzymatically isolated from WT and syndecan-4^−/−^ mice three weeks after AB or SHAB (n = 3 in each group) by cannulating the aorta and retrogradely perfusing with a Ca^2+^-free Tyrode's solution containing 0.05 mg/ml collagenase (Yakult, Tokyo, Japan) for 10 min. The left ventricle was then removed and minced, cardiomyocytes precipitated, and extracellular [Ca^2+^] was gradually increased. Cells were stored at room temperature until use. Cell length and width measurements were determined in a genotype-blinded manner from 2-dimensional images of randomly selected cells.

### Electron microscopy

Two hearts from WT and syndecan-4^−/−^ mice, respectively, obtained three weeks after AB or SHAB were perfused with 2.0% glutaraldehyde buffered in 0.2 M cacodylate at pH 7.4 for 15 min. LV blocks (about 1 mm^3^) were fixed in 2% glutaraldehyde in cacodylate buffer for 2 h, rinsed overnight and transferred to a 1% OsO_4_ solution for 30 min on ice. After rinsing in cacodylate, the specimens were dehydrated in graded ethanols and embedded in Epon. Ultrathin sections (60–100 nm) were stained with uranyl acetate and lead citrate and examined and photographed in a Tecnai G2 spirit BioTWIN 120 kV, LaB6, Transmission Electron Microscope with 4 k Eagle camera (FEI Company, Eindhoven, The Netherlands). All images were examined by two investigators, blinded for mouse identity.

### Histology

Hearts fixed in formalin were processed into paraffin wax. Six 4 µm thick slices were cut along the short axis from the mid-ventricular level of each tissue sample. Slices were stained with hematoxylin-eosin for morphology.

### Immunohistochemistry

The excised hearts were placed in ice-cold phosphate-buffered saline (PBS, pH 7.4), quickly embedded in Tissue-Tek™ O.C.T. compound (Sakura Finetek, Torrance, CA, USA) and frozen in dry ice-cooled isopropanol before storing at −70°C. Five µm thick sections were cut using the Cryostat 1720 from Leitz, Wetzlar, Germany and collected on SuperFrost®Plus slides (Menzel-Gläser, Braunschweig, Germany). Sections were fixed for 5 minutes in acetone at −20°C and air dried. After rinsing in Tris-buffered saline (TBS, pH 7.6) containing 0.05% Tween 20, sections were incubated with the DAKO Peroxidase Blocking Reagent (DAKO, Glostrup, Denmark) for 15 minutes at 37°C followed by TBS rinsing. Sections were then incubated for 30 minutes at 37°C with affinity-purified antibodies (rabbit) against collagen type I (1∶100 dilution) (Rockland, Gilbertsville, PA). After three washes in TBS the primary antibody was labelled for 30 minutes using the DAKO EnVision™ +, Peroxidase, Rabbit K 4010 system and 3,3′-diamino-benzidine tetrahydrochloride as the chromogen. Sections were counterstained with hematoxylin for 1–2 minutes, mounted and examined under a Leitz Aristoplan microscope.

### RNA isolation and real time quantitative PCR

Total RNA was isolated from the LV of mice and LV biopsies from patients using RNeasy mini kit (Cat# 74106, Qiagen Nordic, Norway). RNA quality was determined using 2100 Bioanalyzer (Agilent Technologies, Palo Alto, CA). RNA integrity number (RIN) >7.5 was required for further analysis. Reverse transcription reactions were performed with iScript cDNA Synthesis Kit (Bio-Rad Laboratories, Inc., Hercules, CA). Pre-designed TaqMan assays (Applied Biosystems, Foster City, CA) were used to determine gene expression of α-skeletal actin (Mm00808218_g1), ANP (Mm01255748_g1), BNP (Mm00435304_g1), RCAN1-4 (Mm01213406_m1) and GAPDH (Mm03302249_g1) in WT and syndecan-4^−/−^ mice three weeks after AB or SHAB, and expression of syndecan-4 and RCAN1-4 (Hs01120954_m1) in human myocardial biopsies (Hs01120909_m1). The results were detected on an ABI PRISM 7900 Sequence Detection System (Applied Biosystems).

### Tissue extracts

#### LV tissue extract A

Frozen murine LV tissue was pulverized in a mortar with liquid nitrogen before transfer to lysis buffer (20 mM Hepes, pH 7.5, 150 mM NaCl, 1 mM EDTA, 0.5% Triton) with protease (1 mM PMSF and Complete EDTA-free tablets; Roche Diagnostics) and phosphatase inhibitors (1 mM Na_3_VO_4_, 10 mM NaPP and 50 mM NaF) in a glass tube. The samples were homogenized with a Polytron 1200 and centrifuged at 70 000× g for 60 min at 4°C. Supernatants were collected and stored at −70°C. **LV tissue extract B.** Frozen murine LV tissue or human myocardial LV biopsies were homogenized using a Polytron 1200 in a PBS buffer containing 1% Triton X-100, 0.1% Tween20 and protease (Complete EDTA-free tablets) and phosphatase inhibitors (10 mM NaF and 1 mM Na_3_VO_4_), incubated on ice for 30 min and centrifuged at 14 000–20 000 g for 10 min. The resulting supernatants collected and stored at −70°C. Protein concentrations were determined by Micro BCA Protein Assay Kit (Pierce, Rockford, IL).

### Immunoprecipitation

LV extracts (500 µg tissue extract A) from WT mice 24 h after SHAB or AB were incubated with antibodies (2 µg) and 30 µl protein A/G agarose beads (Santa Cruz Biotechnology, Santa Cruz, CA, sc-2003) overnight at 4°C. Immunocomplexes were washed once for 10 min in immunoprecipitation (IP)-buffer (20 mM Hepes, pH 7.5, 150 mM NaCl, 1 mM EDTA, 1% Triton), four times 10 min in IP-buffer with high salt (1 M) and finally once for 10 min in IP-buffer, boiled in SDS loading buffer and analyzed by immunoblotting. Rabbit IgG (sc-2027) and goat IgG (sc-2028) were used as negative controls. Immunocomplexes from HEK-293 extracts (in presence of 1.5 mM CaCl_2_) were washed three times in lysis buffer (20 mM Hepes, pH 7.5, 150 mM NaCl, 1 mM EDTA, 0.5% Triton). Immunoprecipitates were analyzed by immunoblotting.

### Isolation of neonatal mouse cardiomyocytes

Solutions used; Trypsin solution: 1.11 mg Trypsin powder (Sigma, St.Louis, MO) mixed with 30 ml cold Hanks Balanced Salt Solution (HBSS, Gibco-BRL, Gaithersburg, MD). Collagenase solution: 23.25 mg collagenase Type-2 powder (Worthington Biochemical, Lakewood, NJ) mixed with 100 ml HBSS. Light-medium: 375 ml DMEM (Gibco-BRL), 125 ml M-199 (Gibco-BRL), 5 ml 100× penicillin/streptomycin/glutamine solution (Sigma) and 5 ml 1 M HEPES (Gibco-BRL). Dark medium: Light medium mixed with 25 ml horse serum (Gibco-BRL) and 12.5 ml fetal bovine serum (Gibco-BRL). All solutions were filter sterilized.

The procedure is based on a protocol by Masahiko Hoshijima, University of California, San Diego, CA. In brief, 40–60 neonatal pups were quickly decapitated and their hearts removed and placed in cold HBSS. The LV cut into pieces were digested for 1×6 min and 7×10 min in 18 ml of collagenase solution. Each resulting supernatant was transferred to a 50 ml conical tube with cold dark medium on ice, containing serum to inactivate the collagenase. The pooled supernatants were centrifuged at 124× g for 6 min and the pellet resuspended in warm dark medium. The cells were plated in 150 cm^2^ flasks and incubated at 37°C for 2×75 min for differential plating. The resulting suspension with unattached cardiomyocytes was spun at 124×g for 10 min to pellet cells. After resuspension of pellet in warm dark medium, the cells were counted and plated in 6-well dishes or Omni Trays (Omni Tray Cell Culture Treated w/Lid, Nalge Nunc International, Rochester, NY) coated with 0.2% gelatin (Sigma) added 0.1% fibronectin (Sigma) at a density of 2.5×10^5^ cells/ml, and incubated at 37°C.

### Treating cardiomyocytes with peptides

Isolated neonatal cardiomyocytes were cultured for two days in 6-well plates at a density of 0.6×10^6^ cells/ml. Cardiomyocytes were treated with the arginine-coupled (50 µM) peptide (Genscript Corp.) for 90 min and washed twice with RNase-free PBS before being harvested in IP-buffer (20 mM Hepes, pH 7.5, 150 mM NaCl, 1 mM EDTA, 1% Triton X-100) with protease inhibitors (Complete Mini EDTA-free tablets, Roche) or for quantification of luciferase activity as described above. Harvested protein was analyzed by immunoblotting. Peptide-treatment for longer time-periods have been shown to result in considerable degradation of the peptides [49].

### Cyclic mechanical stretch of neonatal cardiomyocytes

Isolated neonatal cardiomyocytes were plated on collagen I coated silicone membranes at a density of 2.5×10^5^ cells/ml. Cyclic mechanical stretch was induced by stretching cells for 10 min–24 hours (10%, 1 Hz) using the FlexCell Tension System FX-4000 (Dunn Labortechnik, Germany). Cyclosporin A (CsA) (SandImmun Neoral, 500 ng/ml; 586107, Novartis, Switzerland) was used as a calcineurin inhibitor with vehicle serving as control (Cremophore, C5135, Sigma).

### Quantification of 3^H^-leucine incorporation

Isolated cardiomyocytes were left in culture for two-five days in order to induce autonomous hypertrophy [18]. Five µCi/ml 3^H^-leucine (American Radiolabel Chemicals, MO) was added to the medium at day two in culture. The cells were washed 6 times in 95% EtOH before harvested in 0.2 mol/l NaOH at day five in culture. Serum-stimulated cells served as positive control. 3^H^-leucine incorporation was quantified by measuring counts per minute (CPM) in duplicates from each sample at the Wallac Winspectral 1414 Liquid Scintillation Counter (PerkinElmer, MA). Samples were diluted in Pico-Fluor 40 (PerkinElmer).

### Transfection of HEK-293 cells

HEK-293 cells were cultured in DMEM supplemented with 10% fetal calf serum, 100 U/ml penicillin, 1% non-essential amino acids, and maintained in a 37°C, 5% CO_2_ humidified incubator. The HEK-293 cells were transfected with HA-tagged syndecan-4 full length, syndecan-4 S179A, syndecan-4 S179E, His-tagged CnAα cloned into pcDNA 3.1/myc-HIS-A (custom made, Genscript Corporation, Piscataway, NJ) using Lipofectamine 2000 as instructed by the manufacturer (Invitrogen, Carlsbad, CA). After 24 h total lysate (IP-buffer or lysis buffer), or cytoplasmic, nuclear and membrane protein fractions were harvested according to the protocol of the Compartmental Protein Extraction Kit (2145, Chemicon/Millipore, Billerica, MA), and analyzed by immunoblotting.

### Pull-down experiments

Biotinylated peptides (5 µM) were incubated in total heart extract (whole tissue lysate B) from WT mice overnight at 4°C. Monoclonal anti-biotin-conjugated beads were added for 60 min before the bead pellet was washed once for 10 min in IP-buffer, four times 10 min in IP-buffer with high salt (1 M) and finally once for 10 min in IP-buffer. To analyse for a direct interaction, biotinylated peptides (10 µM) were incubated with 10 µg/ml recombinant His-TF-CnA (custom made from Genscript Corporation, Piscataway, NJ) in a 100 µl binding buffer (10 mM NaCl, 20 mM Tris/HCl, pH 8, 6 mM MgCl_2_, 0,1% Triton X-100) containing 1% BSA with gentle rocking for 3 h at 4°C. Twenty five µl monoclonal anti-biotin-agarose (A-1559, Sigma, Saint Louis, MO) was added and incubated with gentle rocking overnight at 4°C. After collection by centrifugation, the beads were washed three times with 1 ml binding buffer. Bound proteins were extracted and boiled with 50 µl 2× SDS sample buffer for five min before analysis by immunoblotting.

### Overlays

CnA overlays were conducted using active recombinant protein expressed in *E. coli* (# 14-446, Upstate, Lake Placid, NY), native CnA purified from bovine (# 14-390, Upstate) or endogenous CnA in heart extract. Syndecan-4 overlays were conducted using a biotin-syndecan-4 (171–198) peptide. Peptide arrays consisting of either the cytoplasmic part of syndecan-4 or the whole of CnA synthesized as partly overlapping 20-mer peptides (Biotechnology Centre, Oslo, Norway) were first blocked in 5% non-fat dry milk or 1% casein in Tris-Buffered Saline Tween 20 (TBST) for 2 h at room temperature. The overlays were performed by incubating overnight at 4°C and then washing the membrane 5 times in cold phosphate-buffered saline with Tween 20 for 5 min at 4°C. These were overlayed with CnA (active recombinant protein (Upstate, Lake Placid, NY), native bovine CnA (Upstate) or endogenous CnA from LV extract) or a biotin-syndecan-4 peptide, respectively. Bound CnA or syndecan-4 was detected by immunoblotting.

### Immunoblot analysis

Cell and tissue extracts and immunoprecipitates were analyzed on 6%, 7.5%, 10%, 15% or 4–20% SDS/PAGE and blotted onto PVDF membranes. The PVDF membranes and peptide arrays were blocked in 5% non-fat dry milk or 1% casein in TBST for 60 min at room temperature, incubated for 1–2 h at room temperature or overnight at 4°C with primary antibodies, washed five times five min in TBST and incubated with a horseradish-peroxidase-conjugated secondary antibody. Blots were developed by using ECL Plus (GE HealthCare, RPN2132). The chemiluminescence signals were detected by Las-1000 or Las-4000 (both Fujifilm, Tokyo, Japan). Actin, GAPDH or Coomassie Brilliant Blue (Bio-Rad, R-250) was used as loading control.

### Antibodies

Immunoblotting and immunoprecipitation were carried out using anti-GSK-3β (1∶1000 dilution, #9315, 27C10), anti-pGSK-3β (1∶1000 dilution, #9336, Ser9), Sapk/JNK (1∶500 dilution, #9252), anti-pSAPK/JNK (1∶500 dilution, #9251, Thr183/Tyr185), anti-p38 MAPK (1∶500 dilution, #9212), anti-pp38 MAPK (1∶500 dilution, #9211, Thr180/Tyr182) all from Cell Signaling Technology, Danvers, MA, anti-Δ-Heparan Sulfate (3G10 epitope, 1∶1500 dilution, #370260, Seikagaku Corporation, Tokyo, Japan), anti-syndecan-4 (1∶200 dilution, sc-15350, used for coimmunoprecipitation as epitope mapping demonstrated recognition of amino acid sequence located to N-terminus of syndecan-4 (Fig. S3A)), anti-pS179-syndecan-4 (1∶200 dilution, sc-22252-R), anti-calcineurin A (1∶200 dilution, sc-6124) all from Santa Cruz, CA, anti-syndecan-4 (1∶2000, #1420, a kind gift from A. Horowitz, Angiogenesis Research Center and Section of Cardiology, Dartmouth Medical School, Lebanon, NH and J. Li, Division of Cardiology, Beth Israel Deaconess Medical Center, Harvard Medical School, Boston, MA, epitope mapping demonstrated specific recognition of amino acid sequence located to the V-region in syndecan-4 (Fig. S3B)), anti-pS179-syndecan-4-HRP (1∶2000, #41144, custom made, Genscript Corporation, Piscataway, NJ), anti-EGFR (1∶500, sc-03, Santa Cruz) anti-actin (1∶500 dilution, sc-8432, Santa Cruz), anti-calsequestrin (1∶2500 dilution, PA1-913, Affinity BioReagents, Golden, CO) anti-PKC-α (1∶200 dilution, sc-208, Santa Cruz, CA), anti-CaM (1∶200, C7055, Sigma), anti-pNFATc4 (1∶200 dilution, sc-32630-R), anti-NFATc4 (1∶200 dilution, sc-13036), anti-GAPDH (1∶500 dilution, sc-20357), anti-histone H4 (1∶500 dilution, sc-8658) all from Santa Cruz, anti-collagen type I (1∶750 dilution, #1310-01, Southern Biotech, Birmingham, AL), anti-collagen type III (1∶3000 dilution, #600-401-105, Rockland Immunochem., Gilbertsville, PA), anti-collagen type VIII (1∶500 dilution, #LC-C46861, LifeSpan Biosciences, Seattle, WA), anti-biotin-HRP (1∶2500 dilution, A-0185) and anti-biotin-conjugated beads (A-1559) both from Sigma. Horseradish peroxidase-conjugated anti-mouse (1∶2500 dilution, NA931V), donkey anti-rabbit IgG HRP affinity purified polyclonal antibody (1∶2500 dilution, NA934V) both from GE HealthCare and anti-goat IgGs (1∶2500 dilution, HAF109, R&D Systems, Minneapolis, MI) were used as secondary antibodies.

### Peptide synthesis

Peptide arrays were synthesized on cellulose paper using a Multipep automated peptide synthesizer (INTAVIS Bioanalytical Instruments AG, Koeln, Germany) as described [50]. Arginine coupled peptides were synthesized and purified to 95% purity (Genscript Corp, Piscataway, NJ) [51]. Biotinylated peptides were purified to 55–95% purity (Genscript Corporation or Biotechnology Centre, Oslo, Norway).

Syn-4 (mem+cyt)-HA-Arg_11_: ERTEVLAALIVGGVVGILFAVFLILLLVYRMKKKDEGSYDLGKKPIYKKAPTNEFYAYPYDVPDYA-R_11_

Arg_9_-Syn-4 (cyt): R_9_-RMKKKDEGSY DLGKKPIYKKAPTNEFYA

Biotinylated-syndecan-4: Biotin-RMKKKDEGSYDLGKKPIYKKAPTNEFYA (97% purity)

Biotinylated-pS179-syndecan-4: Biotin-RMKKKDEG**pS**YDLGKKPIYKKAPTNEFYA (95% purity)

Biotinylated-scrambled-syndecan-4: Biotin-GTKYPKMDRGKLFKYKAKPEDNESAYIK (70% purity) or Biotin-YYKPYFAGDLKKAKTPSNEI (85% purity)

Biotinylated-syndecan-1: Biotin-RMKKKDEGSYSLEEPKQANGGAYQKPTKQEEFYA (55–65% purity)

Biotinylated-syndecan-2: Biotin-RMRKKDEGSYDLGERKPSSAAYQKAPTKEFYA (85–90% purity)

Biotinylated-syndecan-3: Biotin-RMKKKDEGSYTLEEPKQASVTYQKPDKQEEFYA (60–70% purity)

### Densitometric Analysis

Densitometric analysis was performed using Image Gauge 3.46, Science lab 99 (Fujifilm, Tokyo, Japan), Image Quant (Fujifilm), Scion Image (Scion Corporation, Frederick, Maryland) or ImageJ (NIH).

### Equipment and settings used for obtaining and processing images

All figure layouts were made using Photoshop CS2 or CS3 (Adobe Systems Inc., San Jose, CA). Cropping of images was carried out, and labeling and molecular size markers were added. When electrophoretic blots were cropped, care was taken to retain important bands, and at least six band widths above and below the band of interest where possible. Occasionally, brightness and contrast was adjusted equally across the entire image, including controls, in Image Gauge or Photoshop CS2 or CS3.

### Statistics

Data are expressed as group mean ± s.e.m. and analyzed in Statistica 6.0 or GraphPad Prism 5.0. All data were tested for normality before comparisons between groups were made. All *in vivo* and PCR data were examined using one-way ANOVA with adjustment for multiple comparisons using the method of Hothorn et al. [52]. Western data were analyzed using paired or unpaired two-tailed Students *t*-test, or ANOVA with subsequent Newman-Keuls or Bonferroni post hoc test. Differences were considered significant for *p*<0.05.

## Supporting Information

Figure S1 Activation of calcineurin-dependent nuclear factor of activated T-cell (NFAT) signaling in cardiomyocytes in response to cyclic mechanical stretch. (A) Relative NFAT luciferase activity in neonatal cardiomyocytes from NFAT-luciferase reporter mice subjected to cyclic mechanical stretch (MS) (10%, 1 Hz) for 10 min–24 hours (n = 6–19). (B) Relative NFAT luciferase activity in neonatal cardiomyocytes from NFAT-luciferase reporter mice subjected to 24 hrs of cyclic mechanical stretch with and without the calcineurin-inhibitor cyclosporin A (CsA) (n = 8–19).Vehicle (Veh) served as control. Values are mean ± s.e.m.
(PDF)

Figure S2 Activation of nuclear factor of activated T-cell (NFAT)-interacting, pro-hypertrophic signaling pathways in wild type (WT) and syndecan-4^−/−^ (Syn-4^−/−^) mice in response to pressure overload induced by aortic banding (AB). Representative immunoblots and relative quantity of (A) calcineurin (CnA), (B) glycogen synthase kinase (GSK)3-β and phosphorylated GSK3-β (pGSK3-β), (C–D) Akt and phosphorylated Akt (pAKT), (E–F) c-jun N-terminal kinases (JNK)2/3 and phosphorylated JNK2/3 (pJNK2/3), (G–H) extracellular signal regulated protein kinase (ERK)1/2 and phosphorylated ERK1/2 (p ERK1/2) and (I–J) p38 and phosphorylated p38 (pp38) in left ventricles (LV) from WT and Syn-4^−/−^ mice 24 h after AB or sham operation (SHAB) (n = 5–6). A calcineurin- rich cell lysate (Ramos) was used as control for the calcineurin-positive protein band. Vinculin and GAPDH were used as loading control. Values are mean ± s.e.m.
(PDF)

Figure S3 Epitope mapping of syndecan-4 antibodies. Syndecan-4 residues important for antibody binding were identified by overlaying an array of immobilized syndecan-4 20-mer peptides with (A) anti-syndecan-4, (sc-15350), (B) anti-syndecan-4 (#1420), (C) anti-pS179-syndecan-4 (sc-22252-R) or (D) anti-pS179-syndecan-4-HRP (# 41144). Underlined and bold amino acids indicate the core epitope. (A) Anti-syndecan-4 (sc-15350) recognized the amino acid sequence SIRETEVIDPQDLLEGRYFSGALPDD located to the N-terminus of syndecan-4 consistent with the source information from the producer (Santa Cruz) (n = 1). (B) Anti-syndecan-4 ( #1420) strongly and specifically recognized the amino acid sequence KDEGSYDLGKKPIYKKAPTNEFYA located to the cytoplasmic part of syndecan-4. Further mapping revealed that the core epitope was located to the V-region in syndecan-4 (underlined sequence in B) (n = 3). Both pS179-syndecan-4 antibodies (sc-22252-R, (n = 2) and #41144 (n = 3)) recognized pS179-syndecan-4 strongly (right panels in C and D, respectively) compared to the non-phosphorylated syndecan-4 (left panels). Consistent with our findings and according to Genscript, the #41144 antibody is 32 times more specific for pS179-syndecan-4 than non-phosphorylated syndecan-4.
(PDF)

Table S1 Animal characteristics, heart rates, left ventricular pressures and echocardiographic measurements.
(DOC)

Table S2 Myocardial structure and neurohormonal changes.
(DOC)
