# Supplementary figures and images for: Syndecan-4 Is Essential for Development of Concentric Myocardial Hypertrophy via Stretch-Induced Activation of the Calcineurin-NFAT Pathway
Source: PLoS One. 2011 Dec 2;6(12):e28302. doi: 10.1371/journal.pone.0028302 (PMC3229559; doi:10.1371/journal.pone.0028302)

A

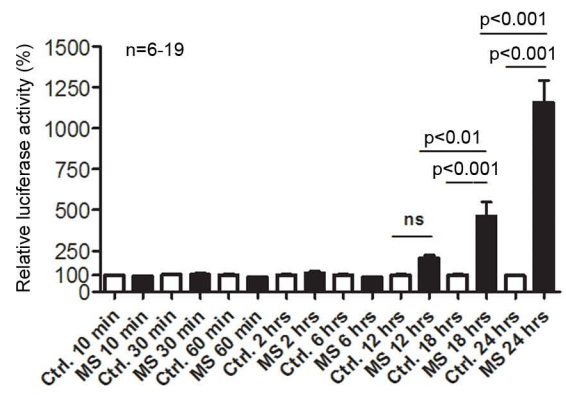

B

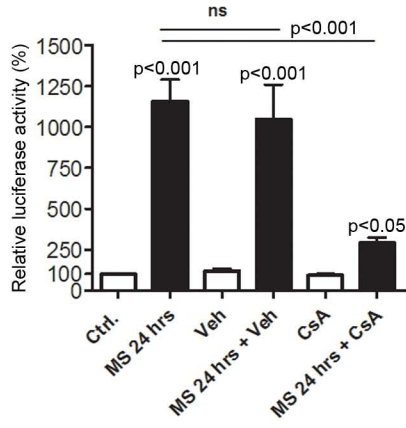

Supplement: Figure S1 — Activation of calcineurin-dependent nuclear factor of activated T-cell (NFAT) signaling in cardiomyocytes in response to cyclic mechanical stretch. (A) Relative NFAT luciferase activity in neonatal cardiomyocytes from NFAT-luciferase reporter mice subjected to cyclic mechanical stretch (MS) (10%, 1 Hz) for 10 min–24 hours (n = 6–19). (B) Relative NFAT luciferase activity in neonatal cardiomyocytes from NFAT-luciferase reporter mice subjected to 24 hrs of cyclic mechanical stretch with and without the calcineurin-inhibitor cyclosporin A (CsA) (n = 8–19).Vehicle (Veh) served as control. Values are mean ± s.e.m. (PDF) [file pone.0028302.s001.pdf]

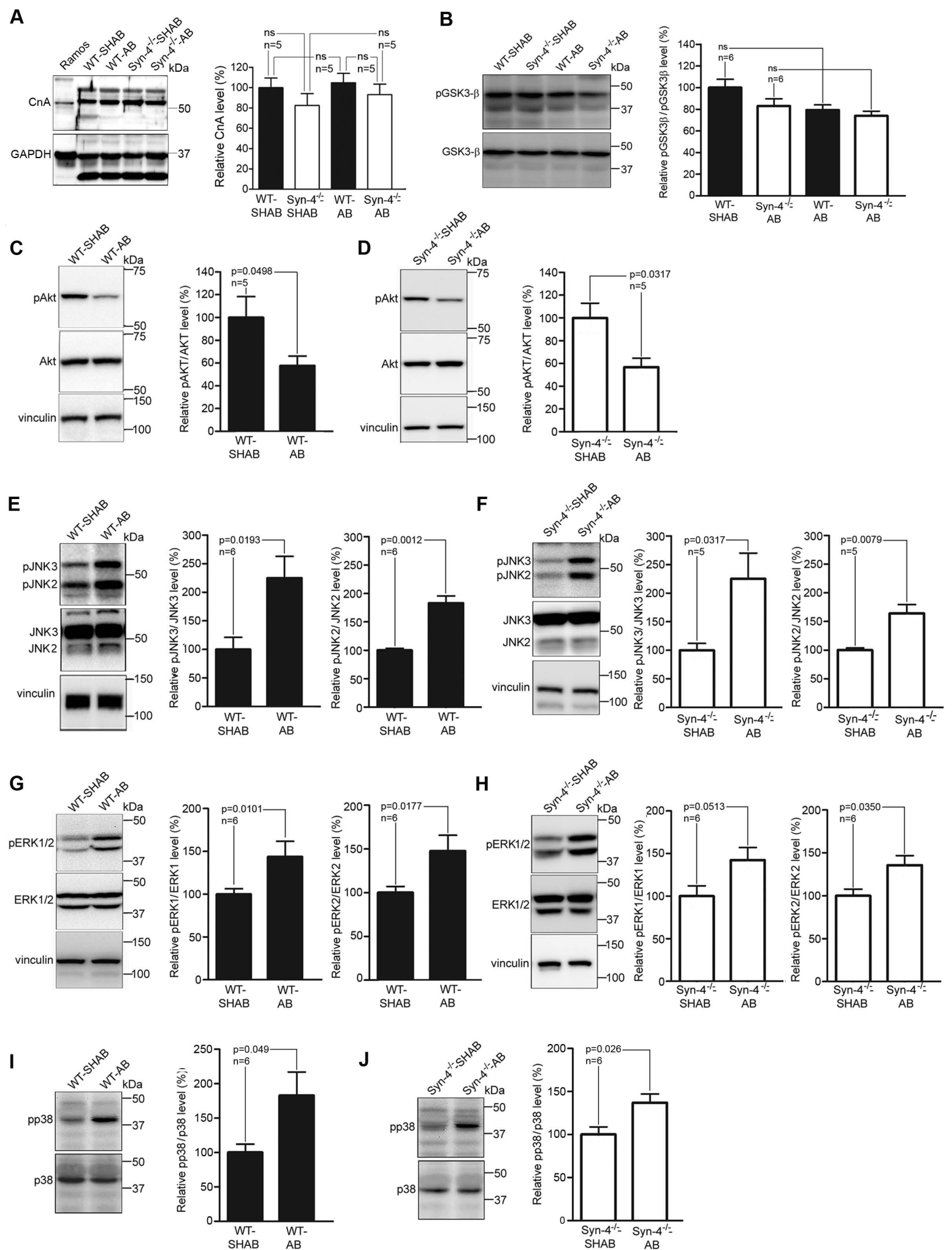

Suppl. Figure S2  
Finsen et al.

Supplement: Figure S2 — Activation of nuclear factor of activated T-cell (NFAT)-interacting, pro-hypertrophic signaling pathways in wild type (WT) and syndecan-4−/− (Syn-4−/−) mice in response to pressure overload induced by aortic banding (AB). Representative immunoblots and relative quantity of (A) calcineurin (CnA), (B) glycogen synthase kinase (GSK)3-β and phosphorylated GSK3-β (pGSK3-β), (C–D) Akt and phosphorylated Akt (pAKT), (E–F) c-jun N-terminal kinases (JNK)2/3 and phosphorylated JNK2/3 (pJNK2/3), (G–H) extracellular signal regulated protein kinase (ERK)1/2 and phosphorylated ERK1/2 (p ERK1/2) and (I–J) p38 and phosphorylated p38 (pp38) in left ventricles (LV) from WT and Syn-4−/− mice 24 h after AB or sham operation (SHAB) (n = 5–6). A calcineurin- rich cell lysate (Ramos) was used as control for the calcineurin-positive protein band. Vinculin and GAPDH were used as loading control. Values are mean ± s.e.m. (PDF) [file pone.0028302.s002.pdf]

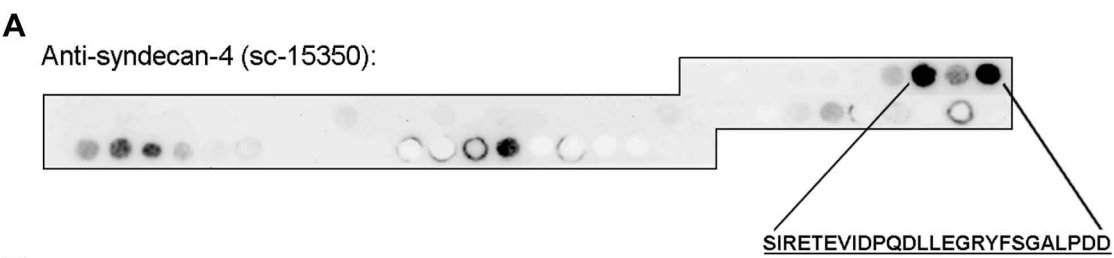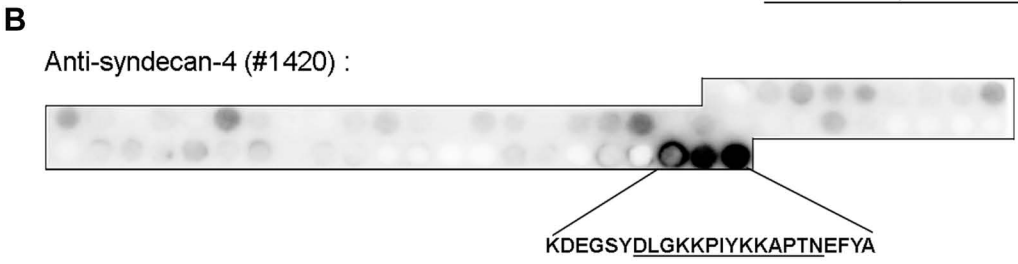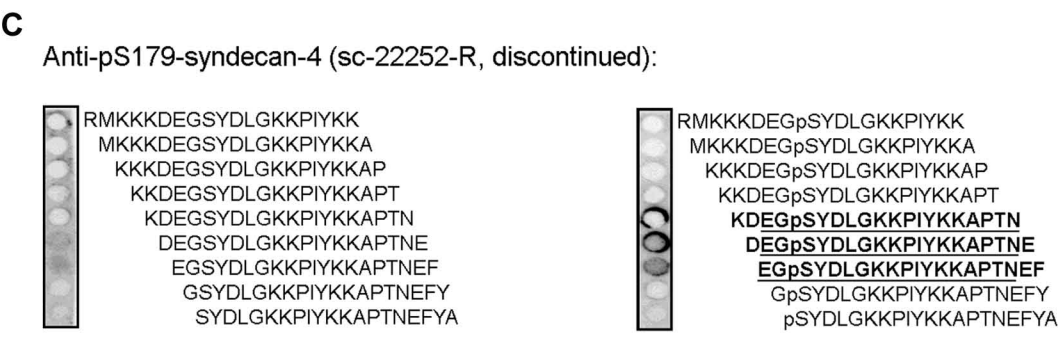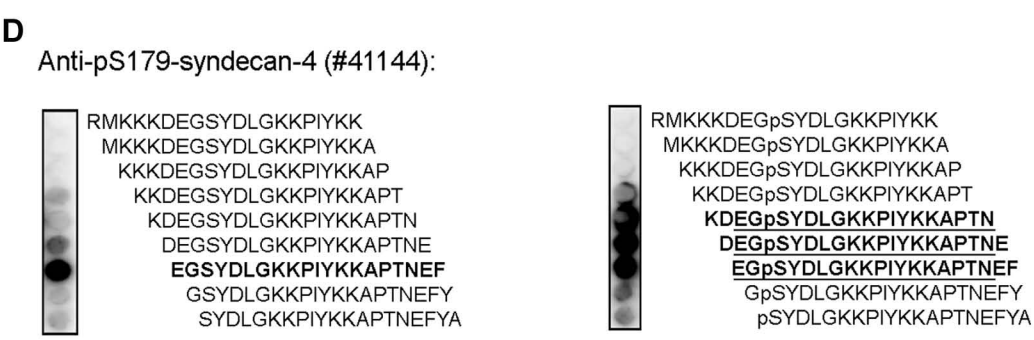

Suppl. Figure S3  
Finsen et al.

Supplement: Figure S3 — Epitope mapping of syndecan-4 antibodies. Syndecan-4 residues important for antibody binding were identified by overlaying an array of immobilized syndecan-4 20-mer peptides with (A) anti-syndecan-4, (sc-15350), (B) anti-syndecan-4 (#1420), (C) anti-pS179-syndecan-4 (sc-22252-R) or (D) anti-pS179-syndecan-4-HRP (# 41144). Underlined and bold amino acids indicate the core epitope. (A) Anti-syndecan-4 (sc-15350) recognized the amino acid sequence SIRETEVIDPQDLLEGRYFSGALPDD located to the N-terminus of syndecan-4 consistent with the source information from the producer (Santa Cruz) (n = 1). (B) Anti-syndecan-4 ( #1420) strongly and specifically recognized the amino acid sequence KDEGSYDLGKKPIYKKAPTNEFYA located to the cytoplasmic part of syndecan-4. Further mapping revealed that the core epitope was located to the V-region in syndecan-4 (underlined sequence in B) (n = 3). Both pS179-syndecan-4 antibodies (sc-22252-R, (n = 2) and #41144 (n = 3)) recognized pS179-syndecan-4 strongly (right panels in C and D, respectively) compared to the non-phosphorylated syndecan-4 (left panels). Consistent with our findings and according to Genscript, the #41144 antibody is 32 times more specific for pS179-syndecan-4 than non-phosphorylated syndecan-4. (PDF) [file pone.0028302.s003.pdf]
