# Supplementary material for: Syndecan-4 Is Essential for Development of Concentric Myocardial Hypertrophy via Stretch-Induced Activation of the Calcineurin-NFAT Pathway
Source: PLoS One. 2011 Dec 2;6(12):e28302. doi: 10.1371/journal.pone.0028302 (PMC3229559; doi:10.1371/journal.pone.0028302)
Supplement: Table S1 — Animal characteristics, heart rates, left ventricular pressures and echocardiographic measurements. (DOC) [file pone.0028302.s004.doc]

**Supporting information**

| **Table S1. Animal characteristics, heart rates, left ventricular pressures and echocardiographic measurements** | | | | | | | | | |
| --- | --- | --- | --- | --- | --- | --- | --- | --- | --- |
|  |  | Un-operated | |  | SHAB | |  | AB | |
|  |  | WT | Syn-4^-/-^ |  | WT | Syn-4^-/-^ |  | WT | Syn-4^-/-^ |
| Characteristics |  |  |  |  |  |  |  |  |  |
|  | n | 6 | 6 |  | 10 | 9 |  | 10 | 10 |
|  | BW, g | 21.8 ± 1.3 | 21.9 ± 1.1 |  | 23.7 ± 0.9 | 25.0± 0.9 |  | 25.7 ± 1.2 | 21.3 ± 0.9* |
|  | TL, mm | 15.9 ± 0.1 | 17.3 ± 0.1* |  | 16.6 ± 0.2 | 17.4 ± 0.1* |  | 17.1 ± 0.2 | 17.5 ± 0.1 |
|  | LVW/TL, mg/mm | 4.45 ± 0.17 | 4.55 ± 0.17 |  | 4.78 ± 0.15 | 4.84 ± 0.24 |  | 7.77 ± 0.28§ | 8.19 ± 0.22§ |
|  | RVW/TL, mg/mm | 1.15± 0.09 | 1.10 ± 0.07 |  | 1.22 ± 0.03 | 1.19 ± 0.05 |  | 1.63 ± 0.12§ | 1.98 ± 0.12*§ |
|  | LW/TL, mg/mm | 8.88 ± 0.27 | 8.49 ± 0.22 |  | 8.48 ± 0.12 | 8.56 ± 0.18 |  | 16.03 ± 2.40§ | 23.98 ± 1.77*§ |
| Echo-2D |  |  |  |  |  |  |  |  |  |
|  | n |  |  |  | 10 | 9 |  | 10 | 10 |
|  | LVILDd, mm/mm |  |  |  | 0.479 ± 0.009 | 0.463 ± 0.008 |  | 0.491 ± 0.008 | 0.532 ± 0.007*§ |
| Echo-M-mode |  |  |  |  |  |  |  |  |  |
|  | n | 6 | 6 |  | 10 | 9 |  | 10 | 10 |
|  | IVSd/TL, mm/mm | 0.044 ± 0.002 | 0.042 ± 0.002 |  | 0.046 ± 0.001 | 0.043 ± 0.001 |  | 0.063 ± 0.002§ | 0.044 ± 0.002* |
|  | IVSs/TL, mm/mm | 0.056 ± 0.004 | 0.050 ± 0.003 |  | 0.055 ± 0.001 | 0.055 ± 0.002 |  | 0.079 ± 0.002§ | 0.049 ± 0.003* |
|  | LVDd/TL, mm/mm | 0.250 ± 0.007 | 0.235 ± 0.006 |  | 0.246 ± 0.004 | 0.240 ± 0.009 |  | 0.243 ± 0.005 | 0.277 ± 0.009*§ |
|  | LVDs/TL, mm/mm | 0.202 ± 0.009 | 0.188 ± 0.004 |  | 0.193 ± 0.006 | 0.189 ± 0.011 |  | 0.193 ± 0.008 | 0.262 ± 0.012*§ |
|  | LVFS, % | 19 ± 3 | 24 ± 5 |  | 22 ± 2 | 22 ± 3 |  | 24 ± 2§ | 8 ± 1*§ |
|  | PWd/TL, mm/mm | 0.040 ± 0.001 | 0.036 ± 0.002 |  | 0.043 ± 0.001 | 0.043 ± 0.001 |  | 0.062 ± 0.002§ | 0.048 ± 0.003* |
|  | PWs/TL, mm/mm | 0.052 ± 0.002 | 0.048 ± 0.002 |  | 0.057 ± 0.002 | 0.057 ± 0.002 |  | 0.079 ± 0.003§ | 0.056 ± 0.002* |
|  | LVEF, % | 40 ± 5 | 41 ± 2 |  | 44 ± 3 | 44 ± 4 |  | 44 ± 3 | 17 ± 2*§ |
|  | LAD/TL, mm/mm | 0.114 ± 0.006 | 0.103 ± 0.005 |  | 0.096 ± 0.004 | 0.098 ± 0.003 |  | 0.148 ± 0.010§ | 0.176 ± 0.006*§ |
|  | AOD/TL, mm/mm | 0.093 ± 0.002 | 0.083 ± 0.003 |  | 0.086 ± 0.002 | 0.087 ± 0.003 |  | 0.089 ± 0.002 | 0.085 ± 0.002 |
| Echo-Doppler |  |  |  |  |  |  |  |  |  |
|  | n | 6 | 6 |  | 12 | 9 |  | 12 | 12 |
|  | HR, beats/min | 436 ± 16 | 479 ± 36 |  | 457 ± 16 | 460 ± 21 |  | 495 ± 14 | 488 ± 12 |
|  | AB V_max_, m/s |  |  |  |  |  |  | 4.3 ± 0.1 | 4.4 ± 0.1 |
|  | Mit PHT, ms | 19.6 ± 2.5 | 21.3 ± 1.5 |  | 20.7 ± 1.3 | 20.9 ± 0.9 |  | 17.2 ± 0.9 | 16.0 ± 0.7§ |
|  | VTI, cm | 4.05 ± 0.40 | 3.71 ± 0.21 |  | 3.86 ± 0.28 | 4.37 ± 0.26 |  | 3.81 ± 0.20 | 2.80 ± 0.22*§ |
|  | CO, ml/min | 20.87 ± 3.08 | 20.84 ± 2.47 |  | 21.39 ± 1.83 | 24.69 ± 2.17 |  | 24.77 ± 2.26 | 17.54 ± 1.55* |
| Catheterization |  |  |  |  |  |  |  |  |  |
|  | n | 9 | 9 |  |  |  |  |  |  |
|  | LVSP, mmHg | 95 ± 2 | 105 ± 4* |  |  |  |  |  |  |
|  | LVEDP, mmHg | 8.9 ± 2.0 | 13.1 ± 2.1 |  |  |  |  |  |  |
|  | LVdP/dt_max_, mmHg/s | 7818 ± 658 | 8843 ± 695 |  |  |  |  |  |  |
|  | LVdP/dt_min_, mmHg/s | 7346 ± 452 | 8691 ± 526 |  |  |  |  |  |  |
| SHAB, 3 week sham-AB operated group; AB, 3 week aorta banded group; WT, wild type mice; Syn-4^-/-^, syndecan-4^-/-^ mice; BW, body weight; TL, tibia length; LVW, left ventricular weight; RVW, right ventricular weight; LW/TL, lung weight; Echo-2D, two-dimentional echocardiography; LVILDd, left ventricular internal longitudinal diameter in diastole; IVSd/s, inter-ventricular septum thickness in diastole respectively systole; LVDd/s, left ventricular diameter in diastole respectively systole; LVFS, left ventricular fractional shortening; PWd/s, posterior wall thickness in diastole respectively systole; LAD, left atrial diameter; AOD, aortic diameter; HR, heart rate; AB Vmax, peak flow over aortic constriction; Mit PHT, mitral pressure half time; LVOT VTI, left ventricular outflow tract velocity time integral; SV, stroke volume; CO, cardiac output; LVSP, left ventricular systolic pressure; LVEDP, left ventricular end diastolic pressure; LVdP/dtmax, maximum rate of left ventricular pressure rise; LVdP/dtmin, maximum rate of left ventricular pressure decline; *, Syn-4^-/-^ significantly different from WT in same group (p < 0.05); §, AB significantly different from SHAB in respective genotype group (p < 0.05); ); catheterization following AB was not performed as the aortic constriction was too tight to allow for retrograde insertion of the catheter. Values are mean ± s.e.m. | | | | | | | | | |
