# Supplementary material for: Syndecan-4 Is Essential for Development of Concentric Myocardial Hypertrophy via Stretch-Induced Activation of the Calcineurin-NFAT Pathway
Source: PLoS One. 2011 Dec 2;6(12):e28302. doi: 10.1371/journal.pone.0028302 (PMC3229559; doi:10.1371/journal.pone.0028302)
Supplement: Table S2 — Myocardial structure and neurohormonal changes. (DOC) [file pone.0028302.s005.doc]

| **Table S2. Myocardial structure and neurohormonal changes** | | | | | |
| --- | --- | --- | --- | --- | --- |
|  | SHAB | |  | AB | |
|  | WT | Syn-4^-/-^ |  | WT | Syn-4^-/-^ |
|  |  |  |  |  |  |
| n | 102 | 112 |  | 120 | 134 |
| Cardiomyocyte width, µm | 14.5 ± 0.3 | 13.7 ± 0.2 |  | 18.4 ± 0.7§ | 14.3 ± 0.3* |
| Cardiomyocyte length, µm | 103.2 ± 1.8 | 101.6 ± 1.6 |  | 111.6 ± 2.0§ | 111.5 ± 1.8§ |
|  |  |  |  |  |  |
| n | 10 | 9 |  | 10 | 10 |
| Collagen type I, % | 100.0 ± 12.3 | 112.9 ± 18.2 |  | 95.4 ± 9.2 | 82.5 ± 12.9 |
| Collagen type III, % | 100.0 ± 6.6 | 85.2 ± 9.5 |  | 173.0 ± 11.1§ | 173.7 ± 11.5§ |
| Collagen type VIII, % | 100.0 ± 6.7 | 95.4 ± 5.0 |  | 142.1 ± 9.7§ | 133.4 ± 10.7§ |
|  |  |  |  |  |  |
| n | 7 | 8 |  | 9 | 12 |
| LV water content, mg | 68.6 ± 1.9 | 64.4 ± 4.8 |  | 103.4 ± 6.6§ | 113.4 ± 3.4§* |
|  |  |  |  |  |  |
| n | 7 | 6 |  | 7 | 7 |
| Angiotensin II, pg/ml | 700 ± 27 | 668 ± 36 |  | 642 ± 23§ | 958 ± 19§* |
| SHAB, 3 week sham-AB operated group; AB, 3 week aorta banded group; WT, wild type mice; Syn-4^-/-^, syndecan-4^-/-^ mice; LV, left ventricle; Collagen assessed by Western blot, values relative to WT-SHAB. *, Syn-4^-/-^ significantly different from WT in same group (p < 0.05); §, AB significantly different from SHAB in respective genotype group (p < 0.05); Values are mean ± s.e.m. | | | | | |
